# Host MKRN1-Mediated Mycobacterial PPE Protein Ubiquitination Suppresses Innate Immune Response

**DOI:** 10.3389/fimmu.2022.880315

**Published:** 2022-05-04

**Authors:** Yafeng Dou, Yan Xie, Lingyun Zhang, Sheng Liu, Dandan Xu, Yuying Wei, Yongshuai Li, Xiao-Lian Zhang

**Affiliations:** ^1^ Hubei Province Key Laboratory of Allergy and Immunology, Department of Immunology, Wuhan University TaiKang Medical School (School of Basic Medical Sciences), Wuhan, China; ^2^ Department of Allergy of Zhongnan Hospital, Wuhan University, School of Medicine, Wuhan, China; ^3^ State Key Laboratory of Virology, Frontier Science Center for Immunology and Metabolism, Wuhan University, Wuhan, China

**Keywords:** *Mycobacterium tuberculosis*, PPE68, MKRN1, ubiquitination, innate immune response

## Abstract

*Mycobacterium tuberculosis (Mtb)*, as an important intracellular pathogen, can invade and survive in macrophages and is capable of escaping the clearance of immune system. Despite decades of research efforts, the precise mechanism of immune escape and the virulence factors encoded by *Mtb* involved remain to be explored. *Mtb*-specific genomic regions of deletion (RD)-encoded proteins and PE/PPE family proteins have been implicated in immune evasion. Here, we screened more than forty RD-encoded proteins which might be involved in facilitating bacterial survival in macrophages, and found that a *Mtb* PPE68/Rv3873 protein, encoded by *Mtb*-RD1, is essential for efficient *Mtb* intracellular survival in macrophages. In terms of mechanism, we found that the ubiquitin ligase (E3) Makorin Ring Finger Protein 1 (MKRN1) of macrophage interacted with PPE68 and promoted the attachment of lysine (K)-63-linked ubiquitin chains to the K166 site of PPE68. K63-ubiquitination of PPE68 further bound src homology 2 domain-containing protein tyrosine phosphatase 1 (SHP1) to suppress K63-linked polyubiquitin chains of tumor necrosis factor receptor-associated factor 6 (TRAF6), and then remarkably suppressed TRAF6-driven NF-κB and AP-1 signaling and TNF-α, IL-6 and NO production. We demonstrate that the K63-linked ubiquitination of PPE68 by MKRN1 contributed to the PPE68-mediated mycobacterial immune escape. Our finding identifies a previously unrecognized mechanism by which host MKRN1-mediated-ubiquitination of mycobacterial PPE protein suppresses innate immune responses. Disturbing the interaction between host MKRN1 ubiquitin system and mycobacterial PPE protein might be a potential therapeutic target for tuberculosis.

## Introduction

Tuberculosis (TB), is a serious infectious disease mainly caused by *Mycobacterium tuberculosis* (*Mtb*). The World Health Organization (WHO) reported that TB patients increased by about 9.87 million a year and about 1.5 million people died in 2021 (1). The emergence of multidrug-resistant TB and co-infection with human immunodeficiency virus (HIV) make TB control even more urgent. Currently, Bacillus Calmette-Guérin (BCG), an attenuated strain of *M. bovis*, is the only TB vaccine available for humans, but this vaccine decreases only childhood TB and gives little protection against adult lung TB; the protection in adults varies from zero to 80% (2). *Mtb* has evolved multiple complex mechanisms that interfere with host cellular processes, such as the blockage of phagosome maturation and autophagosome formation (3), modulation of reactive oxygen and nitrogen species (4), masking, establishing dormancy by manipulating immune responses, altering innate immune cell fate, enhancing granuloma formation, and developing antibiotic tolerance (5). Many efforts have been made to understand how *Mtb* survives in macrophages and *in vivo*. However, the pathogenesis mechanism of TB and the virulence factors encoded by *Mtb* remain to be explored. Therefore, further identification of mycobacterial virulence factors involved in bacterial immune escape will be useful for providing novel strategy or targets for anti-TB drugs and vaccines.

Comparative genomic studies have identified > 100 open reading frames distributed among several virulent *Mtb*-specific genomic regions of deletion (RD) (6). These regions are absent in all avirulent BCG strains but present in virulent H37Rv or some virulent *M. bovis* (6). There are about 16 RDs which encode 129 open reading frames. These RD-encoded proteins might be virulence factors or immune related antigens. *Mtb* RDs encode about 11 PE/PPE proteins related with pathogenesis of *Mtb*, which are characterized by the highly conserved proline-glutamate (PE) or proline-proline-glutamate (PPE) motifs at the N terminus (7). Only a few RD-encoded proteins (e.g., RD1: ESAT-6 (Rv3874) and CFP-10 (Rv3875); RD2: MPT64 (Rv1980c); RD3: EST12 (Rv1579) and RD4: Rv0222) have been characterized and analyzed thus far (8–11). ESAT-6 and CFP-10 contain multiple T cell and B cell epitopes and are widely used in TB diagnosis and vaccine research (12). The functions of most other RD-encoded proteins still remain elusive. Therefore, identifying additional functional RD-encoded proteins might enhances our understanding of the pathogenesis of virulent *Mtb*.

Growing evidence suggests that targeting the host ubiquitin system is a recently emerging aspect of the tactics *Mtb* uses for immune evasion (10, 13). Several E3 ubiquitin ligases, Parkin RBR E3 Ubiquitin Protein Ligase (PARKIN) and SMAD Specific E3 Ubiquitin Protein Ligase 1 (SMURF1) have recently been shown to control ubiquitin targeting of *Mtb* for xenophagy initiation, which was achieved by mediating of lysine (K) 63- and K48-linked ubiquitination of *Mtb*-associated substrates, respectively (14, 15). Makorin Ring Finger Protein (MKRN) orthologs are a novel class of zinc finger proteins, which are conserved in fungi, plants and animals. MKRN1 is the ancestor of this genetic family. Another member, MKRN2, may derive from gene duplication of MKRN1, and is crucial for suppression of the p53/PERP signaling pathway (16, 17). Previous studies have demonstrated that MKRN1 was upregulated during *Mtb* infection and involved in mediating the ubiquitination of *Mtb in vitro* (18, 19), however, its intracellular role during *Mtb* infection has not been investigated.

In the present study, we have screened more than forty RD-encoded proteins which might be involved in facilitating bacterial survival in macrophages, and found that an *Mtb* protein, Rv3873/PPE68-encoded by virulent *Mtb* RD1, is required for efficient *Mtb* intracellular survival in macrophages. We found that the K63-linked ubiquitination of PPE68 at the K166 site mediated by host E3 ubiquitin ligase MKRN1 further inhibited tumor necrosis factor receptor-associated factor 6 (TRAF6)-driven NF-κB/AP-1-TNF-α signaling, and thereby promoted *Mtb* survival in both macrophages and mice.

## Results

### PPE68 Promotes Bacterial Survival and Suppresses Proinflammatory Cytokine and Nitric Oxide Production of Macrophages During Mycobacterial Infection

**Figure 1:**
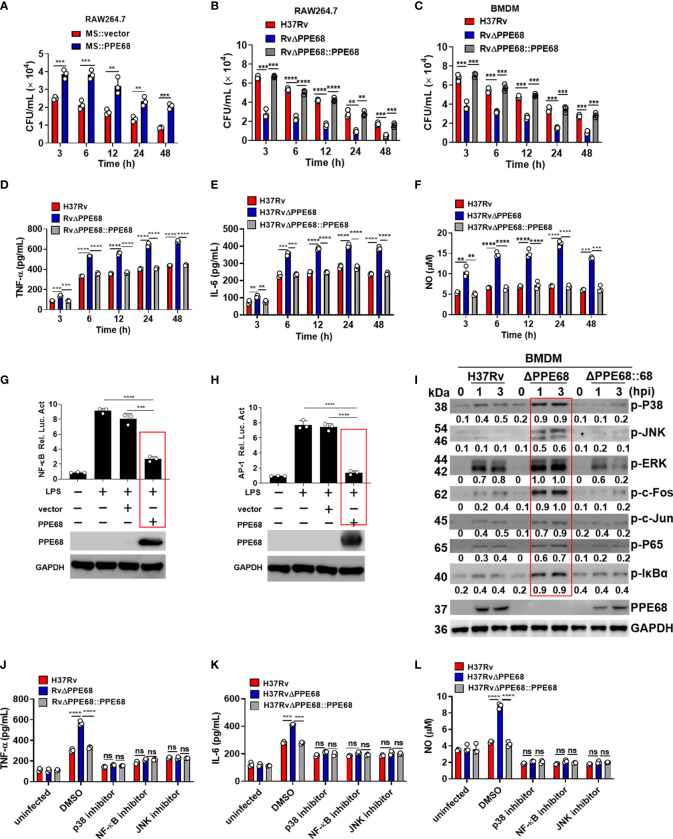
PPE68 promotes mycobacterial survival and suppresses the production of TNF-α, IL-6 and NO in macrophages. **(A-C)** RAW264.7 **(A, B)** or BMDMs **(C)** were infected with MS::vector and MS::PPE68 **(A)** or H37Rv, RvΔPPE68 and RvΔPPE68::PPE68 **(B, C)** at a MOI of 10. The cells were harvested and lysed, and the burdens of intracellular bacteria were determined by CFU assay at various time points. **(D-F)** BMDMs were infected with H37Rv, RvΔPPE68 or RvΔPPE68::PPE68. Secreted TNF-α **(D)** and IL-6 **(E)** protein levels were determined at each indicated time point by ELISA. NO **(F)** production was detected with the Griess assay. **(G, H)** Dual luciferase assay of the effect of PPE68 on NF-κB **(G)** or AP-1 **(H)** activation in RAW264.7 cells. **(I)** Western blot analysis of phosphorylated c-Fos, c-Jun, p65, IκBα, p38 JNK, and ERK and total GAPDH (loading control throughout) expression in BMDMs infected with H37Rv, RvΔPPE68 or RvΔPPE68::PPE68 for the indicated time. Relative densitometry quantification was analyzed based on GAPDH by ImageJ. **(J-L)** BMDMs were pretreated with DMSO, SP600125 (JNK inhibitor, 20 MM), SB203580 (p38 inhibitor, 30 MM), or BAY11-7082 (NF-κB inhibitor, 20 MM) for 1 h followed by infection with different strains (MOI = 10) for 6 h, and then the cells were lysed, and the supernatants were analyzed for TNF-α **(J)**, IL-6 **(K)** and NO **(L)** secretion. Data are expressed as the mean ± SD of three independent experiments for A-H, J-L, and two tailed unpaired t test was used to calculate statistical significance. p > 0.05, not significant (ns); **p < 0.01; ***p < 0.005; ****p < 0.0001.

To identify the H37Rv RD-encoded proteins which are involved in facilitating bacterial survival in macrophages, more than forty recombinant *Mycolicibacterium smegmatis* (MS) strains carrying H37Rv RD-encoded genes were constructed and incubated with bone marrow-derived macrophages (BMDMs) at a multiplicity of infection (MOI) of 10 for 4 h. After 4 h incubation, the infected cells were washed extensively with PBS to remove extracellular mycobacteria. Following this, fresh DMEM supplemented with gentamicin was added to kill extracellular bacteria. At 2 h after infection, cell lysates were plated for bacterial colony-forming units (CFUs) counting. The result showed that an MS strain carrying a RD1-encoded protein PPE68 (MS::PPE68, PPE68 also named as Rv3873 encoded by RD-1, 37.3 kDa) had the highest bacterial CFUs result compared to the other recombinant MS::RD strains (**Supplementary Figures 1A, B** **)**, suggesting that *Mtb* survival in macrophages is enforced by PPE68 after phagocytosis of *Mtb*. Similar growth curves were observed between MS::PPE68 and MS::vector in liquid culture medium (**Supplementary Figure 1C**). We next examined the bacterial survival in MS::PPE68 and MS::vector infected RAW264.7 at various time points. Three hours after infection of RAW264.7 macrophages, bacterial survival of the MS::PPE68 strain infected group markedly increased compared to the MS::vector infected group and this trend continued up to 48 h (**Figure 1A**), suggesting that PPE68 also promotes bacterial survival in macrophages.

PPE68 was confirmed to be present in genomic DNA from the *Mtb* strains H37Rv and H37Ra but not in genomic DNA from BCG, MS, *M. intracellulare*, *M. marinum*, or *M. avium* (**Supplementary Figure 1D**). To further investigate the function of PPE68 during *Mtb* infection, we constructed the PPE68-deficient *Mtb* strain H37RvΔPPE68 (RvΔPPE68) and a rescue strain (RvΔPPE68::PPE68) as described in the Materials and Methods and examined them by Western blot (**Supplementary Figures 2A, B** **)**. The growth curves of RvΔPPE68 and RvΔPPE68::PPE68 displayed no significant difference compared to the wild-type (WT) *Mtb* H37Rv strain (**Supplementary Figure 2C**). RAW264.7 macrophages were infected with H37Rv or RvΔPPE68 for different time course and the bacterial survival was assessed by counting CFUs inside the macrophages. The bacterial CFUs in RvΔPPE68 group was significantly decreased as compared to that of H37Rv or RvΔPPE68::PPE68 groups in RAW264.7 cells (**Figure 1B**). Consistently, a decreased mycobacterial burden was observed in RvΔPPE68-infected BMDMs compared with that of H37Rv or RvΔPPE68::PPE68-infected BMDMs (**Figure 1C**). These data confirm that PPE68 promotes mycobacterial survival in infected macrophages. We did not observe significant difference of cell death among these groups as measured by lactate dehydrogenase (LDH) release assay (**Supplementary Figure 2D**), suggesting that the difference in mycobacterial survival was not due to host cell death. Further investigation showed that the gene deficiency of RvΔPPE68 did not affect the mRNA expression of the upstream Rv3872 gene or downstream Rv3874 gene (**Supplementary Figure 2E**). Above results clearly demonstrate that PPE68 could significantly promote mycobacterial invasion and survival in macrophages during mycobacterial infection.

Bacterial infection triggers the activation of the Toll−like receptor (TLR) signaling pathway, which induces the production of proinflammatory cytokines such as tumor necrosis factor (TNF)-α, interleukin (IL)-6 (20–23). These mediators are critical for the recruitment and activation of immune cells, which work together in response to bacterial infection. TNF-α is a key effector in controlling TB, and loss of TNF signaling causes progression of TB in humans (24). IL-6 is a pleiotropic pro-inflammatory cytokine that is essential for resistance to TB after infection with high doses of intravenous *Mtb* (25). Inducible nitric oxide synthase (iNOS)-mediated nitric oxide (NO) production plays a critical role in host defense against *Mtb* (26, 27). To determine the effect of PPE68 on the production of these proinflammatory cytokines, RAW264.7 cells were infected with the H37Rv or RvΔPPE68 strain for 6 h, and then global gene expression changes were analyzed by RNA sequencing. In total, we identified 211 up-regulated genes and 182 downregulated genes in the RvΔPPE68 strain-infected cells in comparison to the H37Rv group based on RNA-Seq analysis. KEGG enrichment analysis showed that the differentially expressed genes (DEGs) are mainly involved in the TLR signaling pathway, NF-kappa (κ) B signaling pathway, TNF signaling pathway, IL-17 signaling pathway, NOD-like receptor signaling and Herpes simplex virus (HSV)-1 infection (**Supplementary Figure 2F**). We then performed reverse transcription–quantitative real-time polymerase chain reaction (RT-qPCR) experiment to investigate the dynamic expression of proinflammatory cytokines and found that the RvΔPPE68 strain induced more TNF-α (**Supplementary Figure 2G**); IL-6 (**Supplementary Figure 2H**) and iNOS (**Supplementary Figure 2I**) expression than the H37Rv and RvΔPPE68::PPE68 strains in RAW264.7 cells. The production of NO and the proinflammatory cytokines TNF-α, IL-6 were measured at different time points and the results showed that RvΔPPE68-infected BMDMs secreted more TNF-α (**Figure 1D**), IL-6 (**Figure 1E**), and NO (**Figure 1F**) than H37Rv and RvΔPPE68::PPE68-infected BMDMs, suggesting that PPE68 has inhibitory effects on TNF-α, IL-6 and NO expression during mycobacterial infection.

Next, dual luciferase reporter assay was used to determine the effect of PPE68 protein on the NF-κB or AP-1 activation of macrophages. RAW264.7 cells were co-transfected with plasmids encoding PPE68 and the pGL3-NF-κB-luc or pGL3-AP-1-luc reporters. We found that PPE68 significantly suppressed LPS-induced NF-κB (**Figure 1G**) and AP-1 (**Figure 1H**) activation. We then infected BMDMs with H37Rv, RvΔPPE68 or RvΔPPE68::PPE68 to detect the expression of NF-κB/AP-1 pathway signaling molecules. Consistent with the cytokine results, RvΔPPE68 treatment induced higher levels of phosphorylation of p38, JNK, ERK, c-Fos, c-Jun, p65 and IκBα, compared to H37Rv treatment in WT BMDMs, as determined by Western blot analysis (**Figure 1I**). After adding inhibitors of p38, JNK or NF-κB, no significant differences of the secreted TNF-α, IL-6 and NO protein levels of BMDMs were observed among the H37Rv, RvΔPPE68 and RvΔPPE68::PPE68 groups (**Figures 1J-L**). These results clearly demonstrate that PPE68 significantly inhibits *Mtb*-induced TNF-α/IL-6/NO production *via* MAPK and NF-κB/AP-1 signaling pathways.

### PPE68 Is K63 Ubiquitinated by Host MKRN1

**Figure 2:**
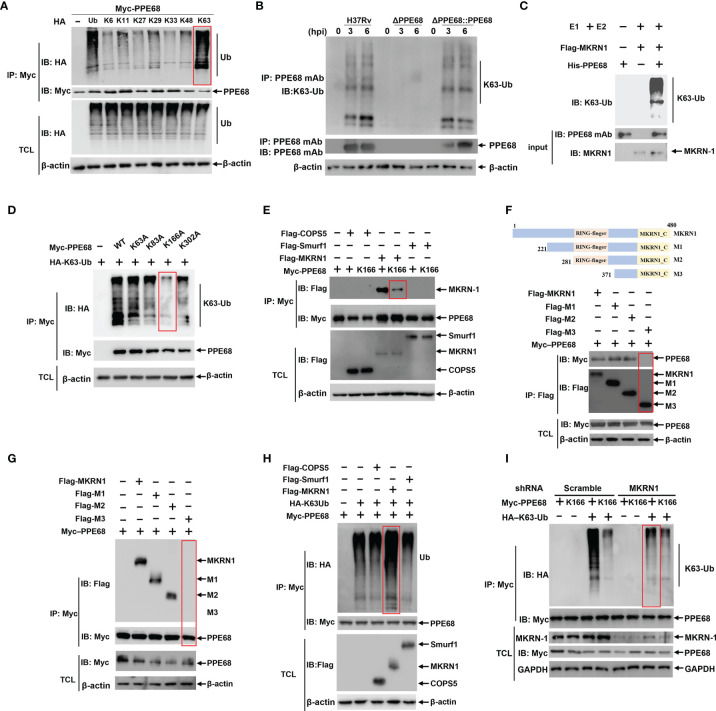
MKRN1 interacts with PPE68 and promotes the attachment of K63-linked ubiquitin chains to K166 of PPE68. **(A, D-I)** HEK293T cells were transfected with the indicated plasmids for 24 h, and then cell lysates were analyzed by immune-precipitation (IP) and Western blot with anti-HA, anti-Myc and anti-Flag antibodies. PPE68 ubiquitination status **(A)**. PPE68 critical ubiquitination site at K166 **(D)**. MKRN1 interacts with PPE68, but not PPE68 K166 mutant **(E)**. MKRN1 critical binding domain for PPE68 **(F, G)**. MKRN1 overexpression promotes ubiquitination for PPE68 **(H)**. PPE68 could not be ubiquitinated after silencing MKRN1 **(I)**. **(B)** PPE68 protein in H37Rv and RvΔPPE68::PPE68 could be ubiquitinated. BMDMs were infected with H37Rv, RvΔPPE68 or RvΔPPE68::PPE68 (MOI=10) for the indicated time course, and then cell lysates were analyzed by IP with an anti-PPE68 mAb and Western blot with an anti-PPE68 mAb and anti-K63 Ub antibodies. **(C)** Western blot analysis of PPE68 ubiquitination *in vitro*. The purified His-PPE68 and Flag-MKRN1 proteins, and the ubiquitin, E1 and E2 ligases were mixed for ubiquitination assay. The mixtures were incubated at 37°C for 90 min and analyzed *via* Western blot with an anti-K63 Ub antibody. TCL, total cell lysates.

Recent reports have shown that host ubiquitination system could modify *Mtb* proteins, thus suppressing anti-*Mtb* innate immune response and promoting mycobacterial pathogenesis (10, 28). We then investigated whether PPE68 could be ubiquitinated in HEK293T cells. There are seven K residues in ubiquitin chain (K6, K11, K27, K29, K33, K48 and K63), which can form polyubiquitin chains. HEK293T cells were co-transfected with Myc-tagged PPE68 and hemagglutinin (HA)-tagged WT ubiquitin (containing all seven lysine residues) or sequential ubiquitin substitution mutants (with only one of the seven lysine residues retained as lysine and the other six replaced with arginine). Co-IP followed by Western blot analysis showed that only the K63 ubiquitin mutant significantly enhanced the ubiquitination of PPE68 (**Figure 2A**), suggesting that PPE68 undergoes K63-linked ubiquitination. In addition, we further determined the increased endogenous K63-linked polyubiquitin on PPE68 in macrophages after infection with H37Rv and RvΔPPE68::PPE68, but not RvΔPPE68 (**Figure 2B**). We also conducted MKRN1-mediated ubiquitination of PPE68 *in vitro* using the Ubiquitinylation kit and found that K63-linked polyubiquitination of PPE68 was achieved only in the presence of MKRN1 (**Figure 2C**). Thus, PPE68 is modified by K63-linked ubiquitination in macrophage.

PPE68 contains four K residues (K63, K83, K166 and K302). To determine which K residue in PPE68 is responsible for the attachment of K63-linked ubiquitination, these four K residues of PPE68 were mutated as alanine (A), respectively, by site-specific mutation. Among these mutants, only PPE68 K166A almost completely abolished K63-linked ubiquitination, suggesting that K166 of PPE68 is the critical site for this ubiquitination (**Figure 2D**). AlphaFold is a system that predicts a protein’s 3D structure from its amino acid sequence (29). We analyzed the structure of PPE68 with the AlphaFold, which clearly shows the presence of four α-helices and six coils (**Supplementary Figure 3A**). These four K residues of PPE68 are indicated with red spheres and the K166 is located on the second coil (**Supplementary Figure 3A**).

We further explored which host E3 ubiquitin ligases might be involved in the ubiquitination of PPE68. MKRN1, COPS5 and SMURF1 were reported as *Mtb*-specific host E3 ubiquitin ligases (15, 19, 30). Using Co-IP followed by Western blot, we demonstrated that MKRN1, not COPS5 and SMURF1, interacted with PPE68 (**Figure 2E**), but K166-mutant PPE68 (K166A) almost completely lost binding to MKRN1 compared to WT PPE68 (**Figure 2E**). We then generated different truncated mutations of Flag-tagged MKRN1, M1 (aa 221 to 480), M2 (aa 281 to 480) and M3 (aa 371 to 480) based on conserved domain of MKRN1 (**Figure 2F**). By using Co-IP (**Figure 2F**) and reversible Co-IP (**Figure 2G**), we found that both M1 (aa 221 to 480) and M2 (aa 281 to 480) were critical for the interaction between PPE68 and MKRN1. Thus, these results indicate that the RING-finger domain of MKRN1 (281 to 371 aa) is essential for the interaction between PPE68 and MKRN1.

Overexpression of MKRN1 significantly promoted WT PPE68 K63-linked ubiquitination (**Figure 2H**), but not of K166-mutant PPE68 (K166A) in HEK293T cells (**Supplementary Figure 3B**). In addition, silencing MKRN1 markedly inhibited K63-linked ubiquitination of PPE68 in HEK293T cells (**Figure 2I**). Thus, host MKRN1 mediates and enhances the K63-linked ubiquitination of the *Mtb* PPE68 at K166 site, and PPE68 binds to MKRN1 through PPE68-K166 site.

### PPE68 Binding With SHP1 Suppresses TRAF6 Ubiquitination and Suppresses TLR2-MyD88-TRAF6-Driven NF-κB/AP-1 Signaling

**Figure 3:**
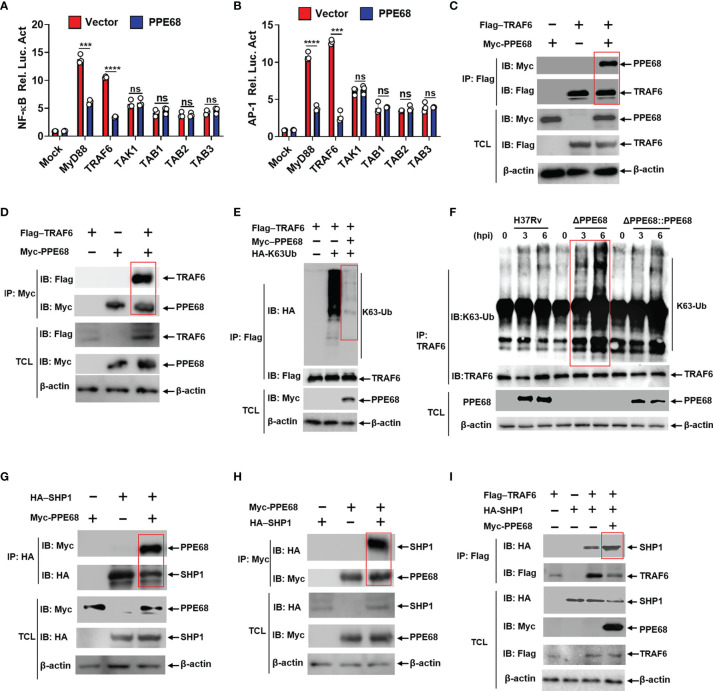
PPE68 interacts with TRAF6 and inhibits TRAF6-NF-κB/AP-1 inflammatory signaling by binding SHP1. **(A, B)** Dual luciferase reporter assay of the effects of PPE68 and various signaling molecules (MyD88, TRAF6. TAK1, and TAB1-3) on the NF-κB **(A)** or AP-1 **(B)** activation in HEK293T cells. **(C-E, G-I)** HEK293T cells were transfected with the indicated plasmids for 24 h, and then cell lysates were analyzed by Co-IP and Western blot. Co-IP and Western blot **(C)** and reversible Co-IP and Western blot **(D)** showed the interaction between PPE68 and TRAF6 **(C, D)**. PPE68 overexpression suppresses TRAF6 K63 ubiquitination **(E)**. Co-IP and Western blot **(G)** and reversible Co-IP-WB **(H)** showed the interaction between SHP1 and PPE68 **(G, H)**. PPE68 promotes the interaction between TRAF6 and SHP1 **(I)**. **(F)** H37Rv or RvΔPPE68::PPE68, but not RvΔPPE68, suppresses TRAF6 K63 ubiquitination. BMDMs were infected with H37Rv, RvΔPPE68 or RvΔPPE68::PPE68 (MOI = 10) for the indicated time; cell lysates were immune-precipitated with an anti-TRAF6 antibody and then analyzed by Western blot with an anti-K63-Ub antibody. TCL, total cell lysates. Data are expressed as the mean ± SD of three independent experiments for A and B, and two tailed unpaired t test was used to calculate statistical significance. *p* > 0.05, not significant (ns); ***p < 0.005; ****p < 0.0001.

PPE68 has been reported to interact with TLR2, inducing IL-10 and MCP-1 expression (31). We also found that PPE68 bound with TLR2 (**Supplementary Figure 3C**). To further explore the effects and molecular mechanism by which PPE68 mediates alteration of various downstream signaling molecules of TLR2, HEK293T cells were co-transfected with eukaryotic expression plasmids encoding PPE68 and various downstream signaling molecules of TLR2, myeloid differentiation factor 88 (MyD88), TRAF6, transforming growth factor-Beta-activated kinase 1 (TAK1) and TGF-beta activated kinase 1 binding protein (TAB)1-3 on the NF-κB/AP-1 signaling pathway and subjected to luciferase reporter assays. We found that PPE68 suppressed MyD88-TRAF6-mediated activation of NF-κB (**Figure 3A**) and AP-1 (**Figure 3B**), suggesting that PPE68 targets TRAF6 for suppression of NF-κB and AP-1 signaling pathway. TRAF6 is widely accepted as an essential regulator of the TLR-mediated inflammatory response (32, 33). We further performed the Co-IP of Myc-tagged PPE68 and Flag-tagged TRAF6 in HEK293T cells. As expected, Flag-TRAF6 could bind Myc-PPE68 (**Figure 3C**). Reversible Co-IP in HEK293T cells also demonstrated the binding between Myc-PPE68 and Flag-TRAF6 (**Figure 3D**). In addition, confocal microscope images showed that colocalization between PPE68 and TRAF6 (**Supplementary Figure 3D**) in the cytoplasm of transfected cells. In addition, we also performed confocal microscopy analysis of infected macrophages to detect the colocalization of *Mtb* with EEA1 (early endosome antigen marker), LAMP1 (lysosomal-associated membrane protein 1, a lysosomal marker), calnexin (an endoplasmic reticulum membrane marker) and GM130 (a Golgi marker) and found that RvΔPPE68 mutant strain had less colocalization with LAMP1 and EEA1 compared to WT H37Rv (**Supplementary Figures 3E-H**). These results suggest that PPE68 may be located in the phagosome and then released from the phagosome into the cytosol, where it encountered cytoplasmic TRAF6. TRAF6 activation is a ubiquitination-dependent process (34). Overexpression of PPE68 significantly inhibited the K63-linked ubiquitination of TRAF6 in HEK293T cells (**Figure 3E**). We also found that PPE68 inhibited endogenous TRAF6 K63 ubiquitination in H37Rv or RvΔPPE68::PPE68 infected BMDMs compared with RvΔPPE68-infected BMDMs (**Figure 3F**). Together, these data suggest that PPE68 inhibits the activation of NF-κB and AP-1 signaling by targeting the TLR2-MyD88-TRAF6-driven NF-κB/AP-1 pathway.

Src homology 2 domain-containing protein tyrosine phosphatase 1 (SHP1) has been reported to bind and inhibit TRAF6 ubiquitination (35). Co-IP (**Figure 3G**) and reversible Co-IP (**Figure 3H**) analysis demonstrated that PPE68 could also interact with SHP1. Therefore, we next examined whether PPE68 affects the interaction between SHP1 and TRAF6. More SHP1 was observed to bound to TRAF6 when PPE68 was overexpressed in HEK293T cells by IP followed by Western blot analysis (**Figure 3I**). By using shTRAF6 and shSHP1 (**Supplementary Figure 4A**), we observed that the inhibitory effect of PPE68 on TNF-α (**Supplementary Figure 4B**), IL-6 (**Supplementary Figure 4C**) and NO (**Supplementary Figure 4E**) expression and the promotive effect of PPE68 on mycobacterial survival in macrophages (**Supplementary Figure 4F**) relied on TRAF6 and SHP1 (**Supplementary Figures 4A-F**). Together, these data strongly demonstrate that PPE68 suppresses TRAF6-driven NF-κB/AP-1-TNF-α/IL-6/NO signaling and promotes *Mtb* survival through interaction with SHP1-TRAF6.

### PPE68 Ubiquitination Suppressed TRAF6-Induced NF-κB and AP-1 Activation

**Figure 4:**
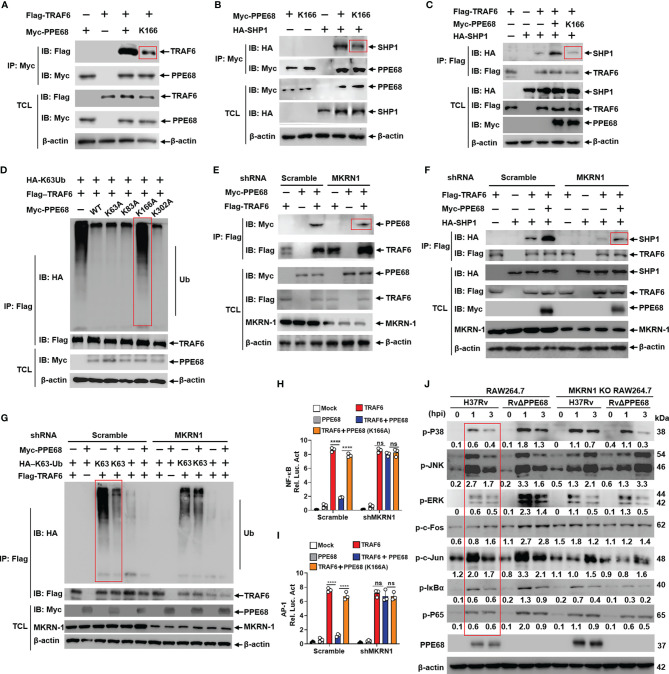
PPE68 K166 ubiquitination interacts with TRAF6 and SHP1, and then inhibits TRAF6-NF-κB/AP-1 signaling. **(A-G)** HEK293T cells were transfected with the indicated plasmids for 24 h, and then cell lysates were analyzed by IP and Western blot with anti-HA, anti-Myc and anti-Flag antibodies. PPE68, but not PPE68 K166 mutant, could interact with TRAF6 **(A)** and SHP1 **(B)**. PPE68, but not PPE68 K166 mutant, overexpression could promote the interaction between TRAF6 and SHP1 **(C)**, and TRAF6 ubiquitination **(D)**. PPE68 interaction with TRAF6 was reduced after MKRN1 silencing **(E)**. After MKRN1 silencing, PPE68 overexpression could not promote the interaction between TRAF6 and SHP1 **(F)**, and could not inhibit TRAF6 K63 ubiquitination, either **(G)**. **(H, I)** After MKRN1 silencing, PPE68, but not PPE68 K166 mutant, could not inhibit TRAF6-driven NF-κB **(H)** and AP-1 **(I)** activation. Dual luciferase reporter assay of the effects of PPE68 or PPE68(K166A) and TRAF6 on the NF-κB **(H)** or AP-1 **(I)** activation in HEK293T cells co-transfected with shMKRN1 and Scramble for 24 (h) **(J)** H37Rv PPE68 inhibits NF-κB/MAPK signaling molecules phosphorylation dependent on MKRN1. Western blot analysis of phosphorylated P38, JNK, ERK, c-Fos, c-Jun, P65, IκBα and P65 and total β-actin (loading control throughout) in WT and MKRN1 KO RAW264.7 cells infected with H37Rv or RvΔPPE68 for the indicated time. Densitometry quantification as shown in panel **(J)** was analyzed based on β-actin by ImageJ. TCL, total cell lysates. The data are expressed as the mean ± SD of three independent replicates for **(H, I)** Two tailed unpaired t test was used to calculate statistical significance. *p* > 0.05, not significant (ns); ****p < 0.0001.

We next examined the effect of the K63-linked ubiquitination of PPE68 on TRAF6-NF-κB/AP-1 signaling. We found that the interaction between PPE68 and TRAF6 or between PPE68 and SHP1 was dramatically decreased when PPE68-K166 was mutated to A (K166A) (**Figures 4A, B** **)**. We also found that the interaction between TRAF6 and SHP1 was dramatically decreased in PPE68-K166A group compared with WT PPE68 group (**Figure 4C**).

We further found that WT PPE68 inhibited, while the PPE68 (K166A) mutant failed to inhibit, the K63-linked ubiquitination of TRAF6 (**Figure 4D**), suggesting that PPE68 K166 ubiquitination promoted the formation of the PPE68-SHP1-TRAF6 complex, and then suppresses TRAF6 K63 ubiquitination and activation.

The interaction between PPE68 and SHP1 or TRAF6 (**Figures 4E, F** **)** was reduced when host E3 ligase MKRN1 was silenced, and PPE68 significantly inhibits the K63-linked ubiquitination of TRAF6 in HEK293T cells (**Figure 4G**), and the inhibitory effect was abolished when MKRN1 was silenced (**Figure 4G**). Dual-luciferase reporter assays showed that PPE68, but not PPE68 (K166A) mutant, efficiently suppressed the TRAF6-mediated activation of NF-κB and AP-1 (**Figures 4H, I** **)**. We then infected WT and MKRN1 knockout (KO) RAW264.7 cells with H37Rv or RvΔPPE68 to detect the expression of NF-κB/AP-1 signaling pathway signaling molecules. Compared to RvΔPPE68 group, H37Rv group has remarkably inhibitory effect on the phosphorylation of ERK, P38, P65, c-fos and IκBα, and slightly inhibitory effect on p-JNK and p-c-Jun, and this inhibitory effect was dependent on MKRN1 (**Figure 4J**).

All above data suggest that PPE68 K166 site ubiquitination mediated by MKRN1 is essential for the interaction between PPE68 and SHP1 or TRAF6, and for the suppression of TRAF6-mediated activation of NF-κB and AP-1 signaling.

Next, we examined whether PPE68 suppresses TNF-α, IL-6 and NO expression and promotes mycobacterial survival dependent on its K166 ubiquitination. Compared with WT RAW264.7 cells, the inhibitory effect on TNF-α, IL-6 and NO expression and the promotive effect on mycobacterial survival by PPE68 were abolished in MKRN1 KO and SHP1 KO RAW264.7 cells infected with H37Rv, RvΔPPE68 or RvΔPPE68::PPE68 (**Supplementary Figures 5A-D**), suggesting that PPE68 suppresses TNF-α, IL-6 and NO and promotes mycobacterial survival dependent on its K166 ubiquitination mediated by MKRN1.

### *Mtb* PPE68 Inhibits TNF-α/IL-6/NO Production and Promotes Mycobacterial Survival *via* PPE68 K166 Ubiquitination in Mice

**Figure 5:**
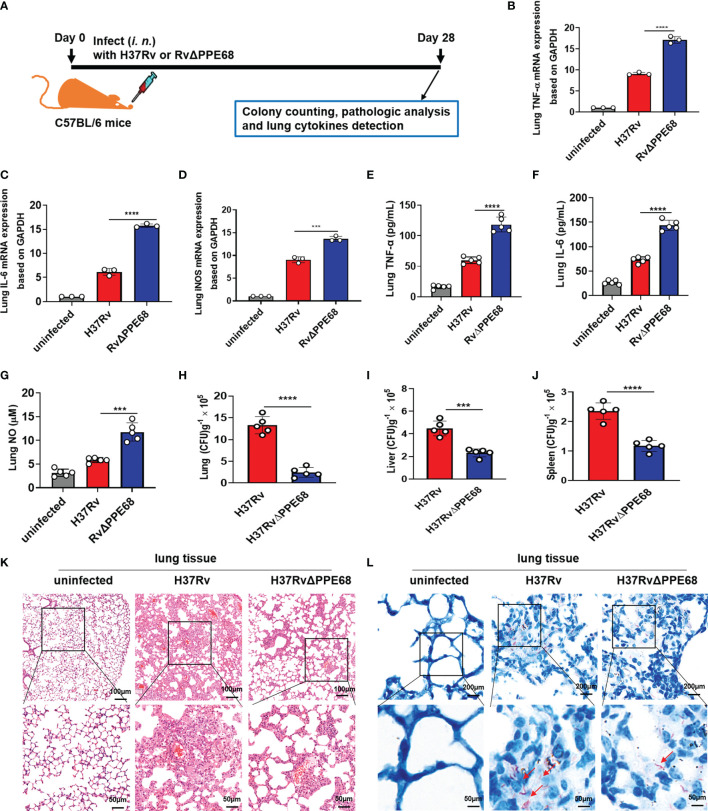
*Mtb* PPE68 inhibits the production of TNF-α, IL-6 and NO and promotes mycobacterial survival in mice. **(A)** Procedure for the mouse *Mtb* infection experiment. **(B-D)** RT-qPCR analysis of lung TNF-α **(B)**, IL-6 **(C)** and iNOS **(D)** mRNA expression of C57BL/6 mice infected (*i.n.*) with 5 × 10^4^ CFUs of H37Rv or RvΔPPE68 (n = 5, each group). **(E-I)** Lung TNF-α **(E)**, IL-6 **(F)**, NO **(G)** concentrations were measured by ELISA, and bacterial burdens in the lungs **(H)**, liver **(I)**, and spleen **(J)** of infected mice were determined by CFU counting on 7H10 agar plates. **(K, L)** Lung tissue sections were analyzed with H&E **(K)** and acid-fast staining **(L)**. The red arrows indicate acid-fast staining-positive bacteria. The data are expressed as the mean ± SD of three independent experiments for B-J, and two tailed unpaired t test was used to calculate statistical significance. ***p < 0.005; ****p < 0.0001.

We further used a mouse infection model to evaluate the role of *Mtb* PPE68 in regulating the production of immunoregulatory molecules *in vivo* (**Figure 5A**). Both the *Mtb* H37Rv and RvΔPPE68 strains were used to infect C57BL/6 mice *via* intranasal (*i.n.*) administration in an acute infection mouse model (9, 36). On day 28 post-infection as shown in **Figure 5A**, RT-qPCR (**Figures 5B-D**), ELISA (**Figures 5E, F** **)** and Greiss assay (**Figure 5G**) showed that the expression of lung TNF-α (**Figures 5B, E** **)**, IL-6 (**Figures 5C, F** **)** and iNOS (**Figure 5D**)/NO (**Figure 5G**) was significantly higher in the RvΔPPE68 infection group than that in the H37Rv infection group.

We further found the number of bacterial colonies in the lungs (**Figure 5H**), liver (**Figure 5I**) and spleen (**Figure 5J**) of the H37Rv group was considerably higher than those in mice infected with the RvΔPPE68 group on day 28 post-infection. A lung histopathological assay also showed that the RvΔPPE68 group had more intact lung tissue structure, fewer lesions with decreased total cellular and neutrophilic infiltration (**Figure 5K**) and a lower abundance of acid-fast bacilli in lung tissue sections than the WT H37Rv group (**Figure 5L**). These results strongly suggest that PPE68 significantly inhibits TNF-α, IL-6 and NO expression, and promotes mycobacterial survival and pathological damage *in vivo*.

To investigate the pathological relevance of the K63-linked ubiquitination of PPE68 during bacterial infection in mice, we challenged WT mice with the indicated strains *via* intranasal (*i.n.*) administration and examined histopathology and bacterial burdens (**Supplementary Figure 6A**). We found that PPE68 had inhibitory effects on MS-induced inflammatory responses (**Supplementary Figure 6B**) by RT-qPCR and lung TNF-α/IL-6 by ELISA (**Supplementary Figure 6C**) and NO levels by Greiss assay (**Supplementary Figure 6D**), respectively, compared to MS::PPE68 (K166A) or MS::vector. Higher bacterial CFUs in the lungs (**Supplementary Figure 6E**), more severe histological damage (**Supplementary Figure 6F**) and more lung acid fast-positive bacilli (**Supplementary Figure 6G**) were observed in MS::PPE68-infected mice than in MS::K166A- and MS::vector control-infected mice. These results clearly suggest that PPE68 suppresses TNF-α, IL-6 and NO-mediated anti-TB immune response and promotes mycobacterial survival vis its K166 ubiquitination *in vivo*.

### *Mtb* PPE68 Inhibits TNF-α/IL-6/NO Production and Promotes Mycobacterial Survival in Mice at Least in Both SHP1 and MKRN1 Dependent Manner

**Figure 6:**
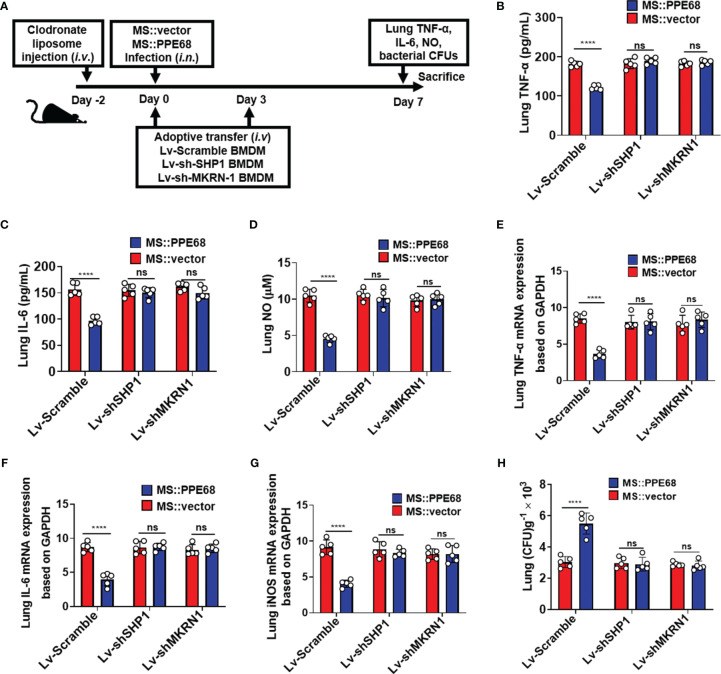
Adoptive transfer of SHP1/MKRN1 knockdown macrophages attenuates the PPE68-mediated suppression of inflammatory cytokine expression and promotion of bacterial survival in mice. **(A)** Procedure for the macrophage adoptive transfer experiment. **(B-D)** Lung TNF-α **(B)**, IL-6 **(C)** and NO **(D)** levels were evaluated by ELISA in the mouse adoptive transfer experiments. **(E-G)** RT-qPCR analysis of lung TNF-α **(E)**, IL-6 **(F)** and iNOS **(G)** mRNA expression in the MS::vector- or MS::PPE68-infected mice after adoptive transfer. **(H)** Mouse lung bacterial burden was determined by CFU counting on 7H10 agar plates (n = 5, each group). The data are expressed as the mean ± SD of three independent experiments for B-H, and two tailed unpaired t test was used to calculate statistical significance. *p* > 0.05, not significant (ns); ****p < 0.0001.

We further adoptively transferred lentivirus-infected BMDMs (as shown in **Figure 6A**), into mice pretreated with clodronate liposome (CL) injection for macrophage depletion. CL injection caused about 87.6% F4/80^+^ macrophages depletion (from 12.1% to 1.5%) (**Supplementary Figure 7A**). Lentivirus containing shSHP1 (Lv-shSHP1) or lentivirus containing shMKRN1 (Lv-shMKRN1) efficiently could knockdown SHP1 and MKRN1 expression in BMDMs (**Supplementary Figure 7B** **upper and lower panel, respectively**). These adoptively transferred mice were infected (i.n.) for 7 days with MS::PPE68 and MS::vector, respectively, for 7 days. Compared to the MS::vector control group, MS::PPE68 group significantly suppressed lung TNF-α/IL-6 by ELISA and NO levels by Greiss assay (**Figures 6B-D**), the lung TNF-α, IL-6 and iNOS mRNA expression by RT-qPCR (**Figures 6E-G**). And MS::PPE68 group also significantly enhanced bacterial survival, as illustrated by the increased CFUs in the mouse lungs (**Figure 6H**), but this effect was abolished in the Lv-shSHP1 or Lv-shMKRN1 BMDM-transferred mice. These data also strongly suggest that PPE68 can suppress inflammatory cytokines expression and promote mycobacterial growth and pathogenesis *in vivo* at least dependent on SHP1 and MKRN1 of macrophages.

## Discussion

During mycobacterial infection, various immune-molecules TNF-α, IL-6, NO and antimicrobial peptides including cathelicidin, defensin, and hepcidin have antimicrobial activities against mycobacteria (37, 38). Increasing evidence suggests *Mtb* modulates the expression of these cytokines and antimicrobial peptides through inhibition of NF-κB and MAPK (28, 38). Immune suppression might be contributed by some PPE family proteins. For example, recent reports indicated that PPE10 and PPE37 could suppress the expression of pro-inflammatory cytokine TNF-α and IL-6 by inhibiting NF-κB and MAPK signaling (39, 40). It was reported that purified PPE68 protein induced IL-10 and MCP-1 secretion and inhibited IL-12p40 expression in a TLR2 dependent manner (31), while the mechanism of immune suppression of PPE68 has not been investigated. Our present study elucidates the mechanism of immune suppression induced by PPE68 at both cellular and animal levels. In this study, we firstly revealed that host E3 ligase MKRN1-mediated PPE68 ubiquitination promoted its binding with SHP1 and TRAF6, thus effectively suppressed TRAF6-induced NF-κB and AP-1 activation by suppressing TRAF6 ubiquitination. PPE68 ubiquitination suppressed TRAF6-driven inflammatory cytokines (TNF-α, IL-6) and NO expression and promoted mycobacterial survival dependent on MKRN1 and SHP1.

MS is usually used as an alternative for *Mtb* in experimental TB, MS has many experimental advantages (biochemically and genetically tractable, nonpathogenic, rapid growth), compared to *Mtb*. Curiously, MS also possesses a highly homologous esxA/esxB genomic locus compared to *Mtb*, which includes PPE68 (41). However, nucleic acid sequence alignment showed that the similarity between the two loci between MS and H37Rv was only 54.1%. Further studies are needed to investigate the function of MS PPE68.

TRAF6 is widely accepted as a signaling adapter molecule common to the IL-1R/TLR family and TNFR superfamily, which is important for proinflammatory cytokine production in response to TLR ligands. K63-linked ubiquitination is critical for TRAF6 activation (42). Ubiquitylated TRAF6 activates its downstream effectors NF-κB and AP-1, leading to the induction of the expression of genes encoding a variety of inflammatory mediators (22). *Mtb* protein Rv0222 can bind to TRAF6 protein and inhibit TLR-mediated macrophage inflammation (10). Rv2626c interacts with TRAF6 and inhibits K63-linked polyubiquitination of TRAF6, which leads to suppression of TLR4 inflammatory signaling in macrophages (43). In the present study, we also demonstrate that PPE68 inhibits TRAF-6 driven inflammatory cytokines signaling of macrophages by inducing TRAF6 deubiquitylation. Thus, it seems that targeting of TRAF6 by microbial proteins to interfere with host immune responses might act as a common and key route for diverse mycobacterial immune escape.

SHP1 is a tyrosine phosphatase that has been reported to play an important role in different diseases. It also has been recognized as a key negative regulator of intracellular signaling (44). A series of studies demonstrated that SHP1 negatively regulates TLR-mediated production of proinflammatory cytokines by suppressing the activation of NF-κB and MAP kinases (45, 46). Recent studies further confirmed several bacterial proteins, such as Tir from enteropathogenic *E.coli* (EPEC), CagA from *Helicobacter pylori* and Rv0222 from *Mtb*, enhanced the association between that SHP1 and TRAF6 (10, 35, 47). However, SHP1 has not yet been reported to be involved in the interaction between mycobacterial PPE and host contributing to the pathogenesis. Here, we found that PPE68 significantly enhanced the interaction between SHP1 and TRAF6, which inhibited K63-linked polyubiquitination of TRAF6 and the inflammatory cytokine production. PPE68 protein was reported to induce chemokine MCP-1 secretion (31), we speculate that it might be involved in recruiting SHP1. However, the mechanism underlying the recruitment of SHP1 by ubiquitylated PPE68 still need further investigation.

Ubiquitin targeting of intracellular bacteria has been reported to play a fundamental role in regulating the host immune response and promoting pathogenesis (48–50). For example, the *Listeria monocytogenes*-secreted listeriolysin O and *Salmonella typhimurium*-secreted effectors, such as SopE and SptP, are also shown to be targets of direct ubiquitination, which are advantageous to the bacterial survival in the cytosol (51). Recently, *Mtb* Rv0222 was reported to undergo K11-linked ubiquitination mediated by the host E3 ubiquitin ligase ANAPC2 in order to suppress the expression of proinflammatory cytokines (10). K11-linked ubiquitination of Rv0222 by ANAPC2 facilitates the recruitment of the protein tyrosine phosphatase SHP1 to the adaptor protein TRAF6, preventing the lysine-63-linked ubiquitination and activation of TRAF6 (10). Although both Rv0222 and PPE68 have been ubiquitinated and thus suppress host innate immune response, they are modified by different host E3 ligases. The UBA domain of the mycobacterial PE_PGRS29/Rv1468c can trigger autophagy by directly binding Ub, which maintains long-term intracellular survival by self-controlling the intracellular bacterial load (13). However, *Mtb* PPE family proteins have not yet been reported to be ubiquitinated by host ubiquitin system.

In the present study, we revealed that the K63-linked ubiquitination of PPE68 protein mediated bacterial immune escape and promoted *Mtb* pathogenesis and survival in both human macrophages in culture and mice. We initially found that host E3 ubiquitin ligase MKRN1, not COPS5 and SMURF1, interacted with PPE68, and PPE68 K166A mutant almost completely lost binding to MKRN1. MKRN1 mediates and enhances the K63-linked ubiquitination of the *Mtb* PPE68 at K166 site. The RING-finger domain of MKRN1 (281 to 335 aa) is essential for the interaction between PPE68 and MKRN1. We also predicted the structure of the PPE68 by using AlphaFold system, which shows that PPE68 has four α-helices and six coils and the K166 is located on the second coil. Future investigation will be needed to identify the structure of the complex between PPE68 and host E3 ubiquitin ligase MKRN1.

MKRN1 functions as an E3 ubiquitin ligase capable of targeting a diverse range of cellular proteins, including hTERT, p53, p21, PPARγ, p14ARF, FADD and PTEN, mediating their ubiquitination and proteasomal degradation (52–54). MKRN1 has been reported to be one of the candidate genes upregulated during *Mtb* infection in cells (18). Based on our present work, we propose that *Mtb* has evolved to utilize the functions of host MKRN1 through PPE68 to support mycobacterial survival and immune escape. However, RvΔPPE68::PPE68 strain was only used in the cell infection model, but not used in the mouse infection model in the present study due to the prevalence of severe acute respiratory syndrome coronavirus 2 (SARS-CoV-2) leading to the closure of ABL3 laboratory. But we have used MS::PPE68, MS::PPE68 (K166A) and MS::vector strains in the mouse infection models to compensate for the limitation. But our results still substantiated our conclusion. In adoptive transfer experiments, macrophage-depleted mice were adoptively transferred with Lv-shMKRN1/Lv-shSHP1-infected BMDMs. Macrophage depletion was attained by clodronate liposome treatment, which primarily depletes macrophages, but also might deplete dendritic cells (DCs) (55). It is still to be explored whether PPE68 exhibits similar effects in DCs.

Based on RNA-Seq analysis, we also observed that DEGs were enriched in HSV-1 infection and NOD-like receptor signaling (**Supplementary Figure 2F**). From RNA-seq analysis we also found that most of 30 genes involved in HSV-1 infection are zinc finger proteins, suggesting that zinc finger proteins might be associated with both HSV-1 and MTB infection. NOD2 recognizes muramyl dipeptide (MDP) from *Mtb*, and MDP is an important contributor to the unusual immunogenicity of mycobacteria but has a limited role in the pathogenesis of *Mtb* infection (56). Although DEGs are enriched in the NOD-like receptor signaling pathway, the NOD2 per se showed no differential expression. Whether PPE68 has effect on MDP in NOD-like receptor signaling and their relation in *Mtb* needs further investigation.

*Mtb* is an extremely successful intracellular pathogen that can be internalized into the phagosome of macrophage. Recently, some mycobacterial factors [such as ESAT-6 of RD1/ESX-1], which mediate mycobacterial escape from the phagosome and translocation to the cytosol, have been identified (9, 57, 58). PE35 and PPE68 are secreted through the ESX-1 system as a heterodimer, and the C-terminal YxxD/E motif of PE35 is required for the secretion (59). Therefore, we speculate that the PPE68 could potentially function similar to another mycobacterial ESX-1 secreted protein ESAT-6, mediating mycobacterial cytosolic translocation and releasing from the phagosome into the cytosol, where it interacted with several host proteins. However, the mechanism of PPE68 involved in the pathway of phagosome release needs to be determined in the future.

**Figure 7:**
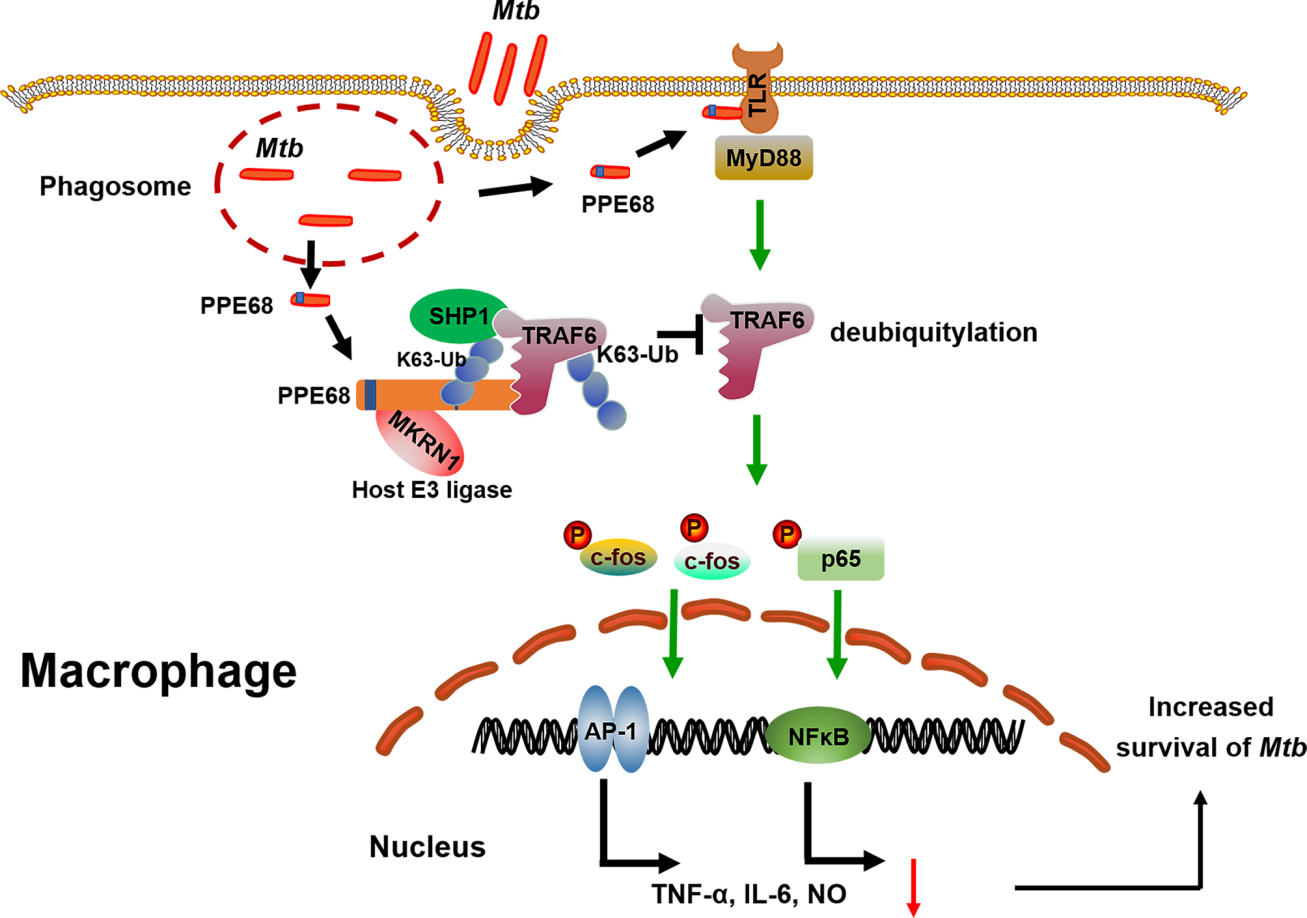
The proposed model of MKRN-1-mediated mycobacterial PPE68 ubiquitination suppressing innate immune responses. PPE68 interacts with MKRN1 and SHP1. Diagram shows the mechanism by which MKRN1-mediated mycobacterial PPE68 K63-linked ubiquitination suppresses host TRAF6 K63 ubiquitination and then inhibits TLR2-MyD88-TRAF6-driven NF-κB and AP-1 inflammatory innate immune responses *via* SHP1 and promotes *Mtb* survival.

Our study still has some limitations. First, this study focused on the function of PPE68 is in macrophages. It is still to be explored whether PPE68 exhibits similar effects in other cells, such as DCs. Second, the mechanism of PPE68 involved in the pathway of phagosome release needs to be determined in the future. Third, future investigation will be needed to identify the structure of the complex between PPE68 and host E3 ubiquitin ligase MKRN1. But our findings clearly reveal a previously unrecognized regulatory role and mechanism for the mycobacterial PPE protein in promoting *Mtb* survival in macrophages and suppressing host innate immune responses (**Figure 7**). Host E3 ligase MKRN1 mediates mycobacterial PPE68 ubiquitination, thus recruits SHP-1 and then suppresses TRAF6 ubiquitination-driven innate immune response *via* a MKRN1-PPE68-SHP1-TRAF6-NF-κB/AP-1-TNF-α/IL-6/NO axis. Our data provides the potential of developing of anti-*Mtb* treatment strategy that targets the interaction between host ubiquitin system and *Mtb* PPE protein.

## Materials and Methods

### Bacterial Strains

*Mtb* H37Rv [American Type Culture Collection (ATCC) 27294] was maintained on Lowenstein-Jensen medium. *Mtb* H37RvΔPPE68 was constructed by Shanghai Gene-optimal Science & Technology Co., Ltd., China. Briefly, the PPE68 gene in the H37Rv genome was replaced with the SacB-hygromycin B cassette by a homologous recombination method. Immunoblot analysis was used to confirm the deletion of PPE68 in the H37Rv strain. The PPE68 gene was cloned into the mycobacterial shuttle vector pMV361, yielding pMV361-PPE68, which was then transformed into the strain H37RvΔPPE68 to create the H37RvΔPPE68::PPE68 strain. *Mycolicibacterium smegmatis* (MS) mc^2^155 (ATCC 700084), *Escherichia coli* (*E. coli*) DH5α (ATCC 25922), and *E. coli* BL-21 (ATCC BAA-1025) were propagated from laboratory stocks (School of Medicine, Wuhan University, Wuhan, China) (9). The mycobacterial strains were grown in Middlebrook 7H9 broth supplemented with 10% oleic acid-albumin dextrose-catalase (OADC) and 0.05% Tween 80 (Sigma-Aldrich) or on Middlebrook 7H10 agar supplemented with 10% OADC and glycerol (5 mL in 1 L of medium).

### Animals and Ethics Statement

C57BL/6 (20–25 g, 6 to 8 weeks, SPF level) mice were used. All mouse experimental protocols were carried out in strict adherence to the Chinese National Guideline for Ethical Review of Laboratory Animal Welfare and approved by the Institutional Animal Care and Use Committee of Wuhan University (No. 18021B) and the Second Military Medical University of Shanghai (No. 18002). All bacterial cultures and animal bacterial challenge experiments were carried out in the Animal Biosafety Level 3 Laboratories (ABL3) of the Wuhan University School of Medicine and the Second Military Medical University of Shanghai. All mice were housed in microisolator cages, fed with standard laboratory diet and water, under controlled conditions of humidity and temperature. The mice were euthanatized with CO_2_ prior to further examination.

### Cell Culture

MKRN1 KO, SHP1 KO RAW264.7 cells were constructed using CRISPR/Cas9 system by Cyagen Biosciences (Guangzhou, China). The complementary oligonucleotide guide RNAs for MKRN1 and SHP1 were listed in **Supplementary Table 1**. RAW264.7, MKRN1 KO, SHP1 KO RAW264.7 cells and HEK293T cells were cultured in Dulbecco’s modified Eagle’s medium (DMEM; HyClone) containing 10% fetal bovine serum (FBS, Gibco) and 100 U/mL of penicillin, and 0.1 mg/mL of streptomycin. BMDMs were differentiated from isolated mouse bone marrow cells by incubation in 10% FBS plus monocyte colony-stimulating factor (M-CSF; 40 ng/ml) for 7 days. Primary cell transfection was performed with NEOFECT™ DNA transfection reagent (Neofect Biotech Co., Ltd., Beijing) according to the manufacturer’s instructions. The transfection of RAW264.7 cells was performed with jetPEI-Macrophage (Polyplus, France).

### Plasmids, Antibodies and Reagents

The PPE68 gene was amplified from the H37Rv genome and cloned into pET28a, pcDNA3.1-Myc-His, and pMV261. Both pGL3-NF-κB-luc, pGL3-AP-1-luc and Renilla were used in the Dual-Luciferase Reporter Assay System (Promega). Short hairpin RNA in pSilencer 1.0-U6 (Ambion Life Technologies, Carlsbad, CA, USA) was used. The cDNA of SHP1 was cloned into pcDNA3.1-HA. The cDNAs of COPS5, Smurf1 and MKRN1 were cloned into pcDNA3.1-Flag. Site-directed mutagenesis of PPE68 was generated by overlap extension PCR. All the plasmids were sequenced at the Beijing Tsingke Biotechnology Co., Ltd. for verification. All plasmids used for this study are listed in **Supplementary Table 2**. Rabbit anti-p-P65 (cat. no. 3033), rabbit anti-p-IκBα (cat. no. 2859), rabbit anti-p-JNK (cat. no. 9251), rabbit anti-p-P38 (cat. no. 4511), rabbit anti-p-ERK (cat. no. 4307), rabbit anti-p-Fos (cat. no. 5348), rabbit anti-p-c-Jun (cat. no. 3207), rabbit anti-TRAF6 (cat. no. 8028), rabbit anti-Calnexin (cat. no. 2679), Alexa Fluor 488-labelled anti-mouse (cat. no. 4408) and Alexa Fluor 594-labelled anti-rabbit (cat. no. 8889) antibodies were all purchased from Cell Signaling Technology (USA). Both anti-Myc mAb (cat. no. TA150121) and anti-MKRN1 mouse mAb (cat. no. CF504092) were purchased from OriGene Technologies, Inc. (China). Rabbit anti-HA-Tag (cat. no. AE036), rabbit anti-GM130 (cat. no. A5344), rabbit anti-LAMP1 (cat. no. A2582), rabbit anti-iNOS (cat. no. A0312), and anti-GAPDH (cat. no. AC002) antibodies were purchased from ABclonal Biotech. Anti-SHP1 (cat. no. 24546-1-AP), anti-EEA1 (cat. No. 28347-1-AP) and anti-β-actin (cat. no. 66009-1-Ig) antibodies were purchased from Proteintech Group (Wuhan, China). Anti-FLAG-Tag mouse mAb (cat. no. 2064) was obtained from Dia-An Biotech (Wuhan, China). SP600125 (JNK inhibitor), SB203580 (p38 inhibitor) and BAY11-7082 (NF-κB inhibitor) were purchased from MedChemExpress (USA). Anti-PPE68 mouse mAb was prepared by Dia-An Biotech (Wuhan, China). Ni-NTA resin was obtained from Qiagen (USA). Mouse M-CSF was obtained from PeproTech (USA). Liposomes and clodronate liposomes (CLs) (cat. no. F70101C-AC-10) were purchased from FormuMax (CA, USA). TNF-α and IL-6 were detected using ELISA kits (Dakewe Biotech, Beijing, China). NO assay kit was purchased from (Beyotime, Shanghai, China).

### Bacterial Intracellular Survival Assay

RAW264.7 cells or BMDMs cultured in DMEM without penicillin-streptomycin were seeded in 6-well tissue culture plates. RAW264.7 cells were infected with MS:vector and MS::PPE68 at a multiplicity of infection (MOI) of 10 at 37°C in 5% CO_2_. After 4 h incubation, the infected cells were washed extensively with PBS to remove extracellular mycobacteria. Following this, fresh DMEM supplemented with gentamicin was added to kill extracellular bacteria. At 3, 6, 12, 24 and 48 h after infection, culture supernatants were harvested, and the macrophages were then washed twice and lysed in sterile PBS containing 0.01% (v/v) Triton X-100. The lysed macrophages were plated on Middlebrook 7H10 agar and the colony forming units were determined as a measure of intracellular survival of recombinant MS. The intracellular survival assay of *Mtb* H37Rv in RAW264.7 and BMDM was performed in a similar manner as described for the MS above. All experiments were performed in triplicate.

### Measurement of TNF-α and IL-6 Cytokine Levels by ELISA

Lung lysates of H37Rv infected mice were harvested and culture supernatants were harvested after infection of macrophages with MS::vector, MS::PPE68, and MS::PPE68 (K166A) or H37Rv, RvΔPPE68 and RvΔPPE68::PPE68 for 3, 6, 12, 24 and 48 h. TNF-α, IL-6 cytokine levels were determined using the mouse Quantikine ELISA kit (Dakewe Biotechnology, Beijing, China). The experiments were performed in triplicate.

### Lactate Dehydrogenase (LDH) Release Assay

The LDH release was measured by a CytoTox 96 NonRadioactive Cytotoxicity Assay (Promega Corporation), according to the manufacturer’s protocol. Culture supernatants were harvested after infection of macrophages with H37Rv, RvΔPPE68 and RvΔPPE68::PPE68 for 3, 6, 12, 24 and 48 h to quantify the release of LDH from the cells. The percentage of specific killing was calculated as follows: specific killing% = (experimental release – spontaneous release)/(total release − spontaneous release) × 100%. The experiments were performed in triplicate.

### Griess Assay for NO Measurement

Culture supernatants were harvested after infection of macrophages with MS::vector, MS::PPE68, and MS::PPE68 (K166A) or H37Rv, RvΔPPE68 and RvΔPPE68::PPE68 for 3, 6, 12, 24 and 48 h. The release NO level was determined using the NO assay kit (Beyotime, Shanghai, China). Supernatants were added to an equal volume of Griess reagent in duplicate on a 96-well plate and incubated at room temperature for 15 min. Absorbance (540 nm) was measured and NO concentrations were estimated using a standard NO curve. The experiments were performed in triplicate.

### Transcriptome Sequencing

Total RNA was extracted using Trizol reagent (Invitrogen, CA, USA) following the manufacturer’s procedure. The total RNA quantity and purity were analysis of Bioanalyzer 2100 and RNA 1000 Nano LabChip Kit (Agilent, CA, USA) with RIN number >7.0. Poly(A) RNA is purified from total RNA (5 μg) using poly-T oligo-attached magnetic beads using two rounds of purification. Following purification, the mRNA is fragmented into small pieces using divalent cations under elevated temperature. Then the cleaved RNA fragments were reverse-transcribed to create the final cDNA library in accordance with the protocol for the mRNASeq sample preparation kit (Illumina, San Diego, USA), the average insert size for the paired-end libraries was 300 bp (± 50 bp). And then the paired-end sequencing was performed on an IlluminaHiseq4000 (LC Sciences, USA).

### Dual-Luciferase Reporter Assay

The Dual-Luciferase^®^ Reporter Assay System (Promega, Madison, United States) was used to measure reporter luciferase activities using a GloMax^®^ 20/20 tube luminometer (Promega, United States). For NF-κB/AP-1 activity detection, RAW264.7 cells were co-transfected with pGL3-NF-κB-luc/pGL3-AP-1-luc, a Renilla luciferase reporter plasmid (pRL-TK, Promega) and pcDNA3.1-Myc-PPE68 plasmid using jetPEI-Macrophage (Polyplus). Then, 24 h post-transfection, cells were treated with LPS (1000 ng/mL, Sigma-Aldrich) to stimulate NF-κB or AP-1 activation.

HEK293T cells were co-transfected with pGL3-NF-κB-luc/pGL3-AP-1-luc, a Renilla luciferase reporter plasmid, pcDNA3.1-Myc-PPE68/pcDNA3.1, and pcDNA3.1-Flag-MyD88/TRAF6/TAK1/TAB1-3, respectively, for 24 h.

Luciferase activities were measured using the Dual-Luciferase Reporter Assay System according to the manufacturer’s instructions (Promega). All data were normalized for transfection efficiency based on Renilla luciferase activity.

### Confocal Microscopy Analysis

To analyze the co-localization of PPE68 and TRAF6, HEK293T cells (3×10^5^) were seeded in confocal dishes (NEST Biological Technology Co., Ltd., Shanghai, China) and transfected with combinations of eukaryotic plasmids (pcDNA3.1-Myc-PPE68, a plasmid encoding Flag-TRAF6). After transfection for 24 h, the cells were stained with anti-Myc, anti-Flag, followed by labeling with anti-Rabbit Alexa Fluor 594 and anti-mouse Alexa Fluor 488-labelled antibodies. DAPI was used to stain nuclei. Confocal images were acquired with a Leica-LCS-SP8-STED confocal system.

For analysis the colocalization of *Mtb* PPE68 in *Mtb*-infected RAW264.7 cells, both WT H37Rv and RvΔPPE68 strains were washed thrice with PBS containing 0.05% Tween-80 by vertexing and resuspended in the buffer, then stained with rhodamine for 30 min at 37°C. These bacteria were again extensively washed and prepared in DMEM medium with 0.05% Tween-80 for infection. RAW264.7 cells were infected with H37Rv-/RvΔPPE68-labeled with rhodamine for 6 h, stained with anti-EEA1, anti-LAMP1, anti-calnexin and anti-GM130 rabbit antibodies, followed by labeling with anti-Rabbit Alexa Fluor 488. DAPI was used to stain nuclei. Confocal images were acquired with a Leica-LCS-SP8-STED confocal system.

### Construction of Short Hairpin RNA (shRNA) Expression Vectors

Construction of shRNA vectors was performed as described previously (60). The pSilencer 1.0-U6 with the U6 promoter was used for gene knockdown. The 21-mer shRNAs against mouse TRAF6 (GenBank accession no. NM_009424.3), mouse SHP1 (GenBank accession no. NM_001077705.2) and human MKRN1 (GenBank accession no. NM_013446.4) mRNAs were designed (**Supplementary Table 1**). Two pairs of coding oligonucleotides were designed for each shRNA based on the targeting sequence. The pairs of oligonucleotides were synthesized, annealed and inserted into the pSilencer vector. Each shRNA sequence contained a 9-bp loop sequence that separated the two complementary domains.

### Reverse Transcription–Quantitative Real-Time Polymerase Chain Reaction (RT-qPCR)

To detect the mRNA expression of TNF-α, IL-6 and iNOS, RAW264.7 cells were seeded in 6-well plates overnight, and then infected with H37Rv, RvΔPPE68 and RvΔPPE68::PPE68 for 3, 6, 12, 24 and 48 h. Total RNA was extracted from the cells using TRIzol reagent (Life Technologies Corporation, USA). The ReverTra Ace-α-First-Strand cDNA Synthesis Kit (Toyobo Biologics Inc., Japan) was used according to the manufacturer’s instructions to synthesize the first-strand complementary DNA (cDNA) from the mRNA in the total RNA sample. The resulting cDNA was stored at −20°C until use in RT-qPCR experiments. The expression of the GAPDH was measured and used as an internal control.

To detect the mRNA expression of Rv3872, Rv3873 and Rv3874, total RNA was isolated from the H37Rv RvΔPPE68 and RvΔPPE68::PPE68 strains using Trizol reagent. All RNA samples were treated with TURBO DNA-free kit (Ambion, Grand Island, NY, USA) to remove DNA contamination. cDNA was synthesized using ReverTra Ace-α-First-Strand cDNA Synthesis Kit. The expression of the 16S RNA was measured and used as an internal control.

The RT-qPCR reactions were performed in triplicate and run on an ABI StepOnePlus (Applied Biosystems) using the standard cycling conditions. Quantification of transcriptional level was calculated according to the 2^−ΔΔCt^ method.

### Immunoprecipitation (IP) and Western Blot Analysis

For IP and Western blot assay, transfected 293T cells or infected RAW264.7/BMDMs were lysed in RIPA lysis buffer with the protease inhibitor phenylmethylsulfonyl fluoride (PMSF). The cell lysates were incubated with anti-Flag plus protein A + G magnetic beads or anti-TRAF6 plus protein A/G magnetic beads followed by extensive washing with RIPA lysis buffer. The immunoprecipitates were analyzed by SDS-PAGE and blotted with the indicated antibodies. RAW264.7 or BMDMs were infected with H37Rv RvΔPPE68 and RvΔPPE68::PPE68 strains for different time course, respectively, cells were harvested and lysed to prepare total protein extracts. The phosphorylation expression of proteins related to NF-κB and MAPK signaling pathways was determined by Western blot.

### Ubiquitination Assay

HEK293T cells were transfected with pcDNA3.1-Myc-PPE68 or Flag-TRAF6 and the indicated HA-tagged ubiquitin plasmids (HA-Ub, HA-K6, HA-K11, HA-K27, HA-K29, HA-K33, HA-K48 or HA-K63) for 48 h. TRAF6 or PPE68 was immunoprecipitated with anti-Flag/anti-Myc antibody and blotted with anti-HA-tag/anti-specific ubiquitin antibodies.

### *In Vitro* Ubiquitination Assay

*In vitro* ubiquitination assay was performed using the Ubiquitinylation Kit (ENZO Life Sciences, Farmingdale, NY) according to the manufacturer’s instructions. His-PPE68 was expressed and purified from *E. coli* BL21-transformed with pET-28a-PPE68-6 × His with Ni-NTA agarose. MKRN1 was expressed and purified from HEK293T cells-transfected with pcDNA3.1-Flag-MKRN1 with anti-Flag plus protein A + G magnetic beads. The Ni-NTA-agarose or Flag-MKRN1-magnetic beads were washed three times with PBS plus 0.05% Tween-20. Flag-MKRN1-magnetic beads were incubated with 1 × ubiquitinoylation buffer, 2.5 mM dithiothreitol, 1 × Mg-ATP solution, 100 nM human E1, 2.5 mM human E2 (UbcH13) and 2.5 mM biotinylated ubiquitin in the reaction buffer. The reaction started with the addition of PPE68 and then incubated at 37°C for 90 min. The reactions were stopped by the addition of loading buffer and analyzed by SDS–PAGE followed by Western blot using anti-K63 ubiquitin antibody.

### Mouse Infection Experiments

For mouse infection experiments, bacteria were dispersed into single-cell suspensions by sonication with BACspreader™ 1100 (TB Healthcare, China) before infection. Each mouse (C57BL/6 mice pretreated with isoflurane anesthesia) was intranasally (*i.n.*) infected with 5×10^4^ CFUs H37Rv or H37RvΔPPE68. On day 28-post-infection, the bacterial numbers in the organs of infected mice were assessed by plating organ homogenates on Middlebrook 7H10 agar and cultured for 3 weeks (61–63). Lung tissues were used for RT-qPCR and ELISA to detect cytokines expression, and for Ziehl–Neelsen acid-fast staining and hematoxylin and eosin (H&E) analysis.

In the MS infection model, C57BL/6 mice were intranasally (*i.n.*) infected with MS::Vector, MS::PPE68 and MS::PPE68 (K166A) with 1×10^7^ CFUs per mouse. The bacterial loads in the lungs of infected mice were assessed. Lung tissues were retained for RT-qPCR and ELISA to detect cytokines expression, and for Ziehl–Neelsen acid-fast staining and H&E analysis.

### Lentivirus Production and Infection

pLKO lentiviral vectors were used for gene knockdown in the adoptive transfer experiment. The shRNA sequences for the pLKO lentiviral vector constructions are listed in **Supplementary Table 1**. Lentiviral vector pLKO.1-shSHP-1 or pLKO.1-shMKRN1 and helper vectors psPAX2 and pMD2.G were co-transfected into HEK293T cells by NOEFECT transfection reagent. The culture supernatants were collected 48 h after transfection and filtered with 0.45 Mm nitrocellulose filter. The collected lentiviruses were used to infect mouse BMDMs for 48 h in presence of 8 µg/ml Polybrene, and cell lysates were analyzed by Western blot. The lentivirus-infected BMDMs were then used for the adoptive transfer experiment.

### Adoptive Transfer of BMDMs and *In Vivo* Mouse Model

For mouse macrophage adoptive transfer experiments (64), C57BL/6 mice (pretreated with isoflurane anesthesia) were intravenously (*i.v.*) injected with clodronate liposomes (CLs) or liposome controls (200 µL per mouse) to deplete macrophages. The depletion efficiency of lung macrophage was evaluated by flow cytometric analysis. On days 2 and 5 post-injection, mice were adoptively transferred with WT, Lv-shSHP-1 or Lv-shMKRN1 BMDMs (5×10^6^ cells per mouse). Two days after injection with CL or liposome controls, the mice were intranasally (*i.n.*) challenged with 1×10^7^ CFUs of MS::vector or MS::PPE68. Seven days after infection, the mice were euthanized, lung TNF-α, IL-6 and NO concentration, lung TNF-α, IL-6 and iNOS mRNA expression were determined by ELISA and RT-qPCR, respectively, and lung bacterial CFUs were determined on Middlebrook 7H10 agar plates.

### Flow Cytometry

Lungs were excised and digested enzymatically at 37˚C for 30 min in PBS with 5% FBS, 3 mg/mL collagenase type IV (Worthington), and 20 U/mL DNase (Roche). The digested tissues were then filtered through 70 µm nylon filters (BD Biosciences), and then cells were washed with sterile PBS. Single cell suspensions were stained with F4/80-APC antibody. Then, the cells were washed twice with PBS and analyzed by flow cytometry with a Beckman CytoFLEX FCM (CA, United States) and data were analyzed using FlowJo Software (Ashland, OR).

### Statistical Analysis

All experiments were repeated at least three times with consistent results. All statistical analyses were performed using GraphPad Prism 8. For comparison between the different groups, a two tailed unpaired t test was used to calculate statistical significance. *p* values < 0.05 were considered to be statistically significant (*p <0.05, **p <0.01, ***p <0.005, ****p< 0.0001).

## Data Availability Statement

The datasets presented in this study can be found in online repositories. The names of the repository/repositories and accession number(s) can be found below: National Genomics Data Center (https://ngdc.cncb.ac.cn/); CRA004222.

## Ethics Statement

The animal study was reviewed and approved by the Institutional Animal Care and Use Committee of Wuhan University (No. 18021B) and the Second Military Medical University of Shanghai (No. 18002).

## Author Contributions

X-LZ designed and supervised the research. YD, LZ, SL, DX, YW, and YL conducted the experiments. YD, YX, and LZ analyzed the data. YD and X-LZ wrote the manuscript, and X-LZ revised the manuscript. All authors contributed to the article and approved the submitted version.

## Funding

This project was supported by grants from the National Grand Program on Key Infectious Disease of China (2017ZX10201301-006 and 2012ZX10003002-015), the National Key R&D Program of China (2018YFA0507603), the National Natural Science Foundation of China (91740120 and 22077097), the National Outstanding Youth Foundation of China (81025008), Hubei Province’s Outstanding Medical Academic Leader Program (523-276003), Hubei Province Key R&D Program (2020BCB020), Research and Innovation Team Project of Hubei Provincial Health Commission (WJ2021C002), the Medical Science Advancement Program (Basic Medical Sciences) of Wuhan University (TFJC 2018002), the Fundamental Research Funds for the Central Universities and Foundation Committee Innovation Group Project (21721005).

## Conflict of Interest

The authors declare that the research was conducted in the absence of any commercial or financial relationships that could be construed as a potential conflict of interest.

## Publisher’s Note

All claims expressed in this article are solely those of the authors and do not necessarily represent those of their affiliated organizations, or those of the publisher, the editors and the reviewers. Any product that may be evaluated in this article, or claim that may be made by its manufacturer, is not guaranteed or endorsed by the publisher.
