# Supplementary material for: Host MKRN1-Mediated Mycobacterial PPE Protein Ubiquitination Suppresses Innate Immune Response
Source: Front Immunol. 2022 May 4;13:880315. doi: 10.3389/fimmu.2022.880315 (PMC9114769; doi:10.3389/fimmu.2022.880315)
Supplement: Supplementary file 1 [file DataSheet_1.docx]

**SUPPLEMENTARY MATERIAL**

**Supplementary Figure 1.**

**
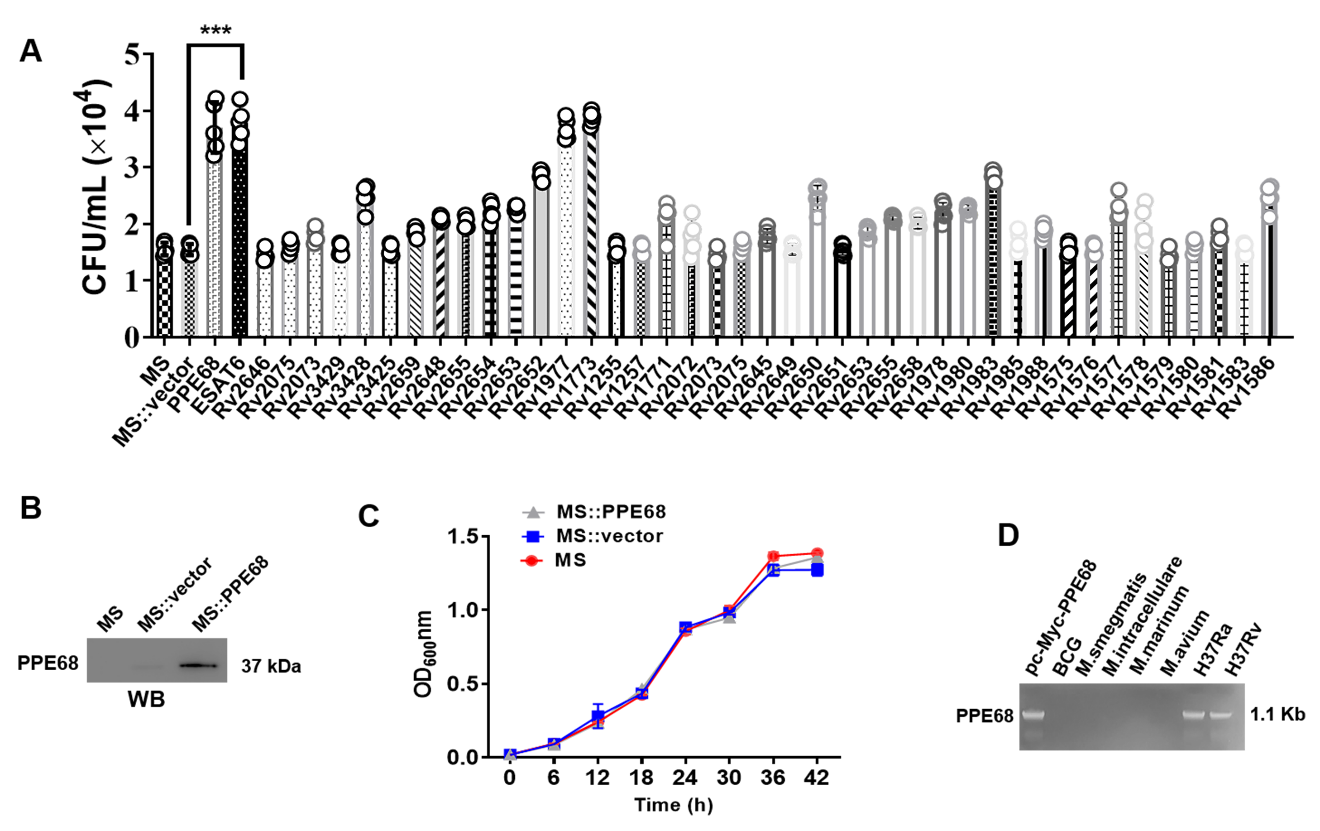
**

**Supplementary Figure 1.** **Survival ability of macrophages by different recombinant MS strains containing RD genes expression plasmids and PPE68 distribution in different mycobacterial strains. (A)** Bacterial colony count assay for invasion assay of macrophages by using different recombinant MS strains containing RD genes expression plasmids (pMV261-RDs). **(B)** Western blot analysis of the MS::PPE68 recombinant strains using an anti-PPE68 mAb. **(C)** The growth kinetics of the indicated recombinant MS strains. **(D)** Identification of PPE68 gene expression in different mycobacterial strains by PCR**.** The data are expressed as the mean ± SD of three independent experiments for **A**, and two tailed unpaired t test was used to calculate statistical significance. *p* > 0.05, not significant (ns); ****p* < 0.005.

**Supplementary Figure 2.**

**
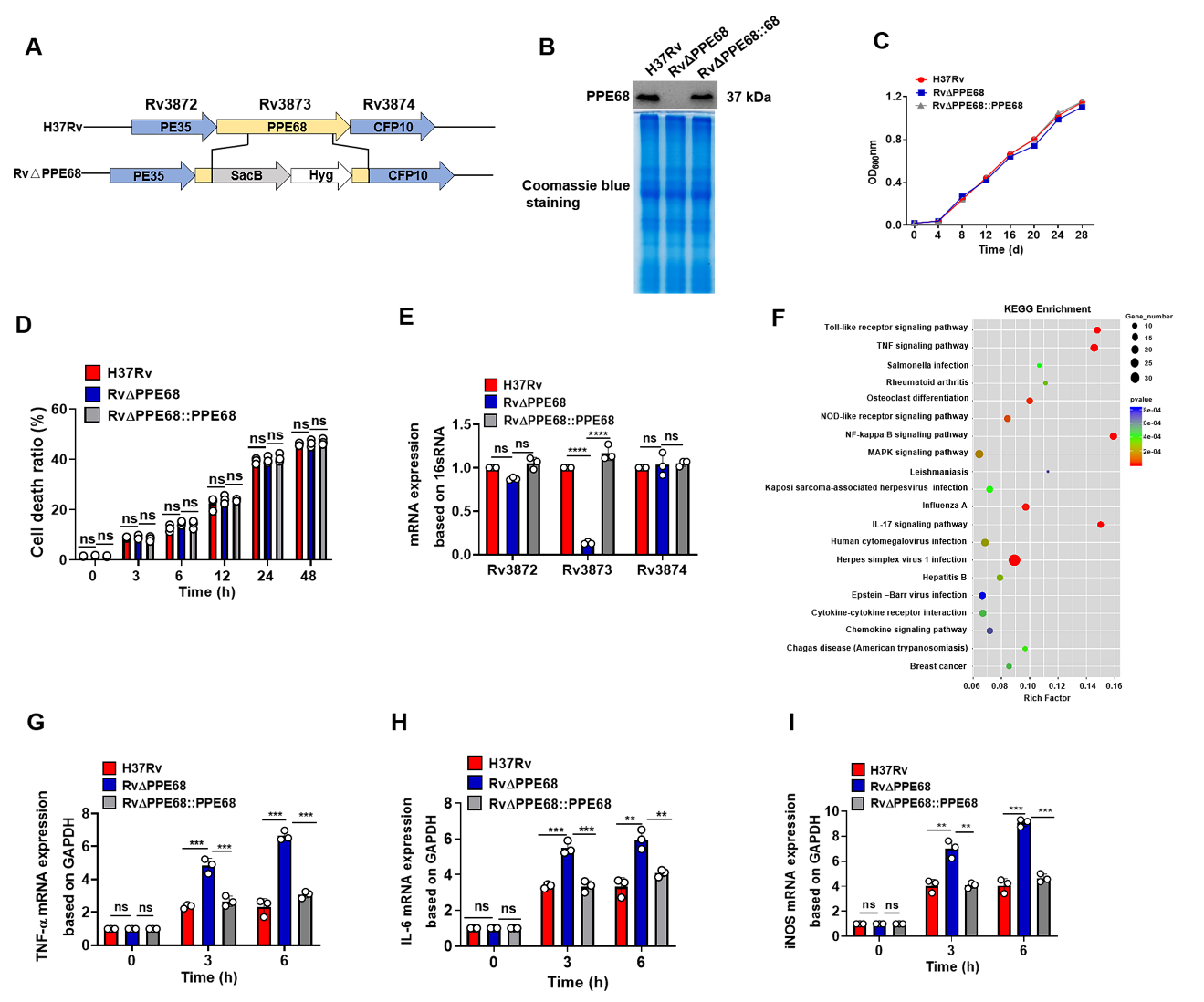
**

**Supplementary Figure 2. Construction and characterization of H37Rv PPE68 mutant strain and effects of PPE68 on cell viability and inflammatory cytokine expression. (A)** Illustration of construction of H37RvΔPPE68 strain. **(B)** Western blot analysis of PPE68 expression using whole bacterial lysates of the H37Rv, H37RvΔPPE68 and H37RvΔPPE68::PPE68 strains. The equality of each loading quantity was assessed by coomassie blue staining. **(C)** The growth kinetics of the three indicated strains. **(D)** Lactate dehydrogenase release assay for cell death analysis. **(E)** RT-qPCR analysis of gene expression of Rv3873, its upstream Rv3872, or its downstream Rv3874 in H37Rv, RvΔPPE68 or RvΔPPE68::PPE68 strains. **(F)** KEGG pathway enrichment analysis of RNA-seq-identified differentially expressed genes. **(G-I)** RT-qPCR analysis of TNF-α (**G**), IL-6 (**H**) and iNOS (**I**) expression at each indicated time point in BMDMs infected with H37Rv, RvΔPPE68 or RvΔPPE68::PPE68, respectively. The data are expressed as the mean ± SD of three independent experiments for **D, E** and **G-I**, and two tailed unpaired t test was used to calculate statistical significance. *p* > 0.05, not significant (ns); ***p* < 0.01; ****p* < 0.005; *****p* < 0.0001.

**Supplementary Figure 3.**


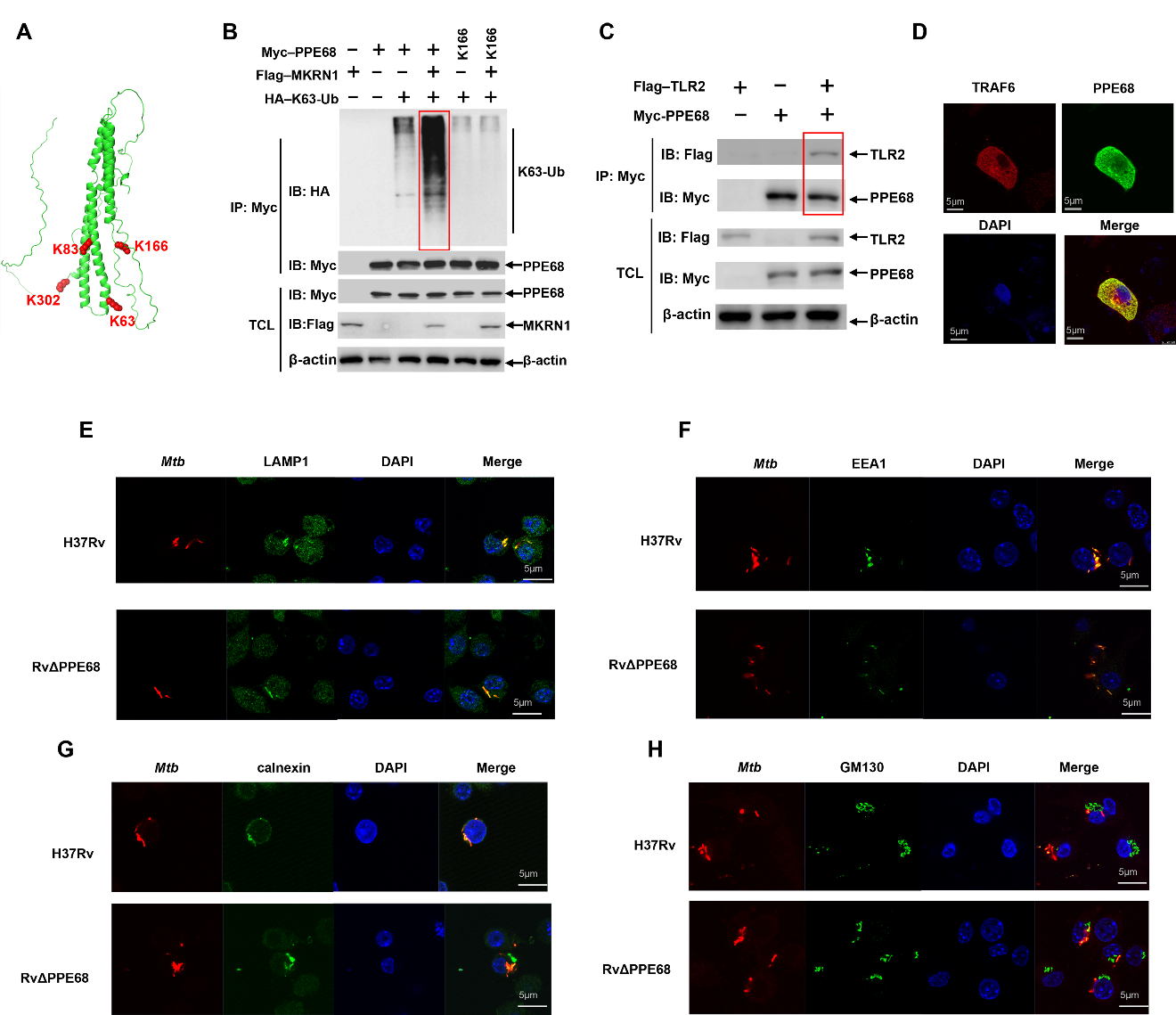


**Supplementary Figure 3. Structure prediction and localization of PPE68. (A)** Graphical representation of K residues (red) location in the AlphaFold-predicted three-dimensional structure of *Mtb* PPE68. (**B** and **C**) HEK293T cells were transfected with the indicated plasmids for 24 h, and then cell lysates were analyzed by immune-precipitation (IP) and Western-blot with anti-HA, anti-Myc and anti-Flag antibodies. MKRN1 overexpression promotes ubiquitination for PPE68, but not for PPE68 K166 mutant (**B**). Co-IP-Western-blot showed the interaction between PPE68 and TLR2 **(C)**. **(D) T**he co-localization of PPE68 and TRAF6 was analyzed by confocal microscopy. HEK293T cells were transfected with pcDNA3.1-PPE68 and Flag-TRAF6 for 24 h. Scale bar, 5 μm. **(E-H)** Confocal microscopy analysis for colocalization of *Mtb* with EEA1 (**E**), LAMP1 (**F**), calnexin (**G**) and GM130 (**H**) in RAW264.7 cells. RAW264.7 cells were infected with the WT H37Rv and RvΔPPE68 mutant strain at MOI = 10 for 6 h and then probed using anti-EEA1, anti-LAMP1, anti-calnexin and anti-GM130 antibodies (green). *Mtb* strains (red) were prestained with rhodamine before infection. Scale bars, 5 μm.

**Supplementary Figure 4.**

**
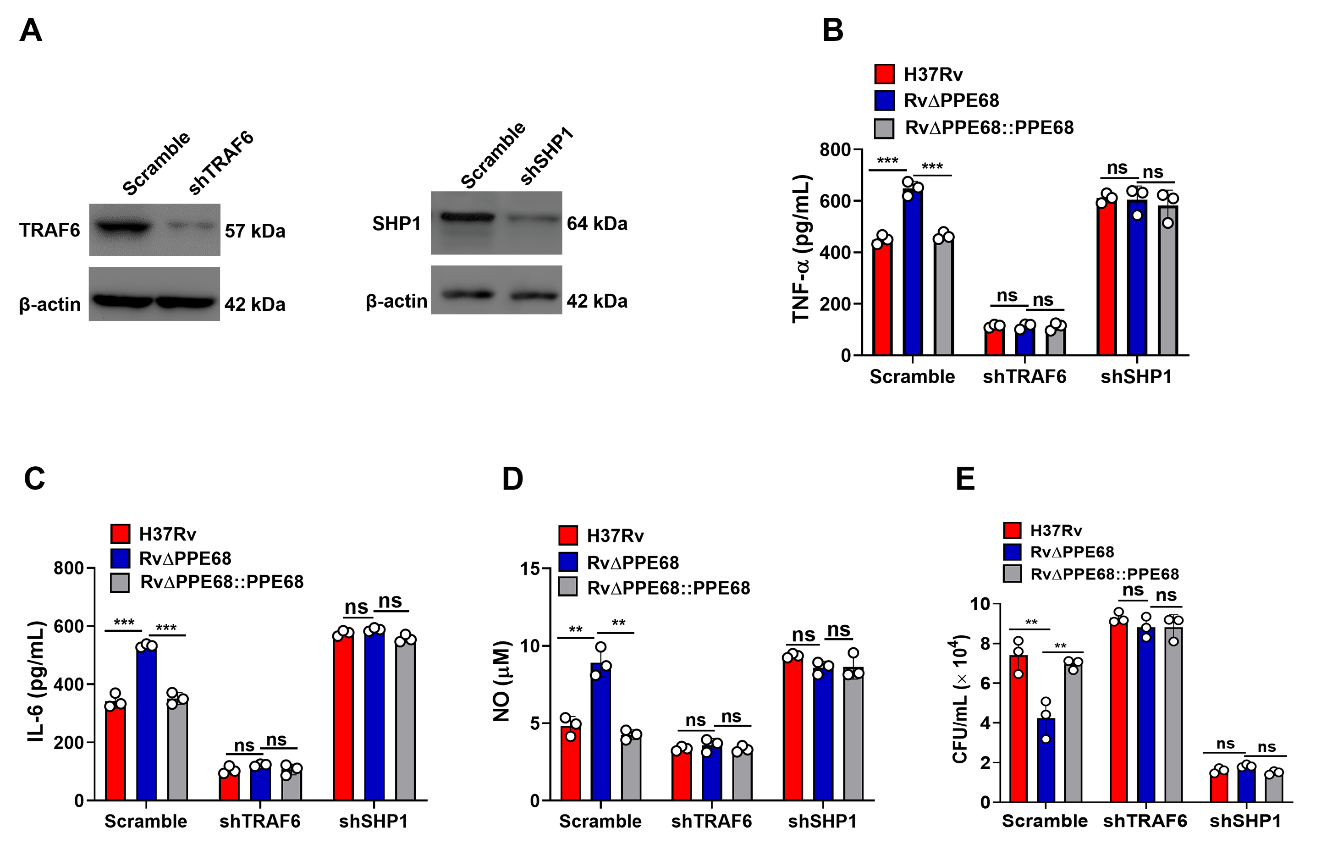
**

**Supplementary Figure 4. Inhibitory effect of PPE68 on TRAF6-NF-κB/AP-1 inflammatory signaling is TRAF6 and SHP-1 dependent. (A)** Western-blot analysis of shTRAF6 (Left) and shSHP1 (Right) silencing efficiency. TRAF6 (Left) and SHP1 (Right) expression with anti-TRAF6 or anti-SHP1 antibodies in RAW264.7 cells after transfection with pSilencer1.0-U6-shRNA-TRAF6/SHP1 or control vector. **(B-E)** PPE68 inhibits inflammatory cytokine production and secretion, and promotes bacterial survival. RAW264.7 cells were transfected with the control vector or shTRAF6/shSHP1 for 24 h followed by H37Rv, RvΔPPE68 or RvΔPPE68::PPE68 infection (MOI = 10) for 6 h. The supernatants were collected for TNF-α **(B)**, IL-6 **(C)** and NO **(D)** secretion analysis and the intracellular bacteria were enumerated **(E)**. The data are expressed as the mean ± SD of three independent experiments for **B-E**, two tailed unpaired t test was used to calculate statistical significance. *p* > 0.05, not significant (ns); **p* < 0.05; ***p* < 0.01; ****p* < 0.005; *****p* < 0.0001. **Supplementary Figure 5.**


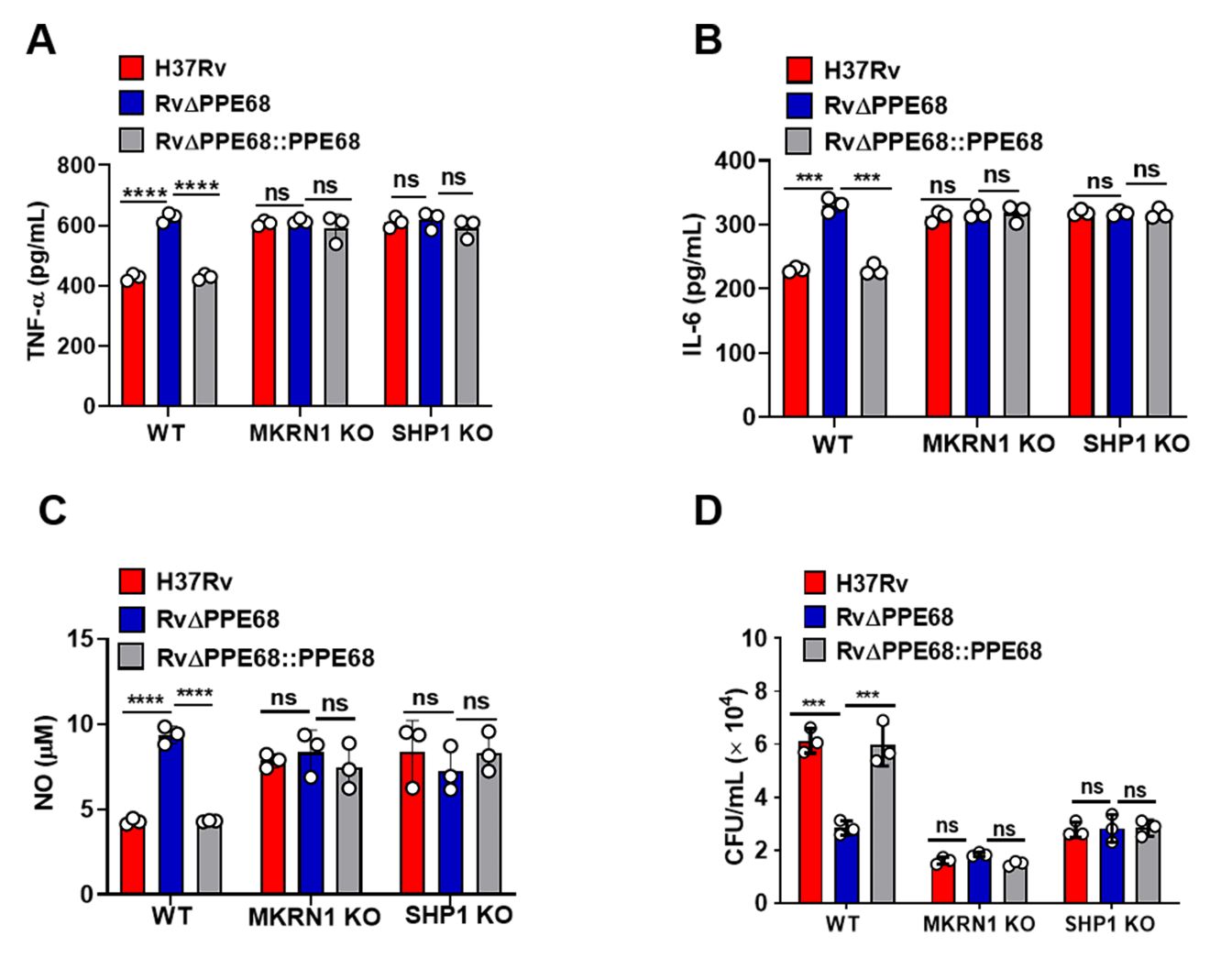


**Supplementary Figure 5. PPE68 inhibits inflammatory signaling and promotes mycobacterial survival dependent on MKRN1 and SHP1 of macrophages.** (**A-D**) WT, MKRN1 KO or SHP1 KO RAW264.7 cells were infected with H37Rv, RvΔPPE68 or RvΔPPE68::PPE68 for 6 h, the cell supernatant was detected for the production of TNF-α (**A**), IL-6 (**B**) and NO (**C**), and intracellular bacteria were enumerated (**D**). The data are expressed as the mean ± SD of three independent experiments for **A-D**, two tailed unpaired t test was used to calculate statistical significance. *p* > 0.05, not significant (ns); ****p* < 0.005; *****p* < 0.0001.

**Supplementary Figure 6.
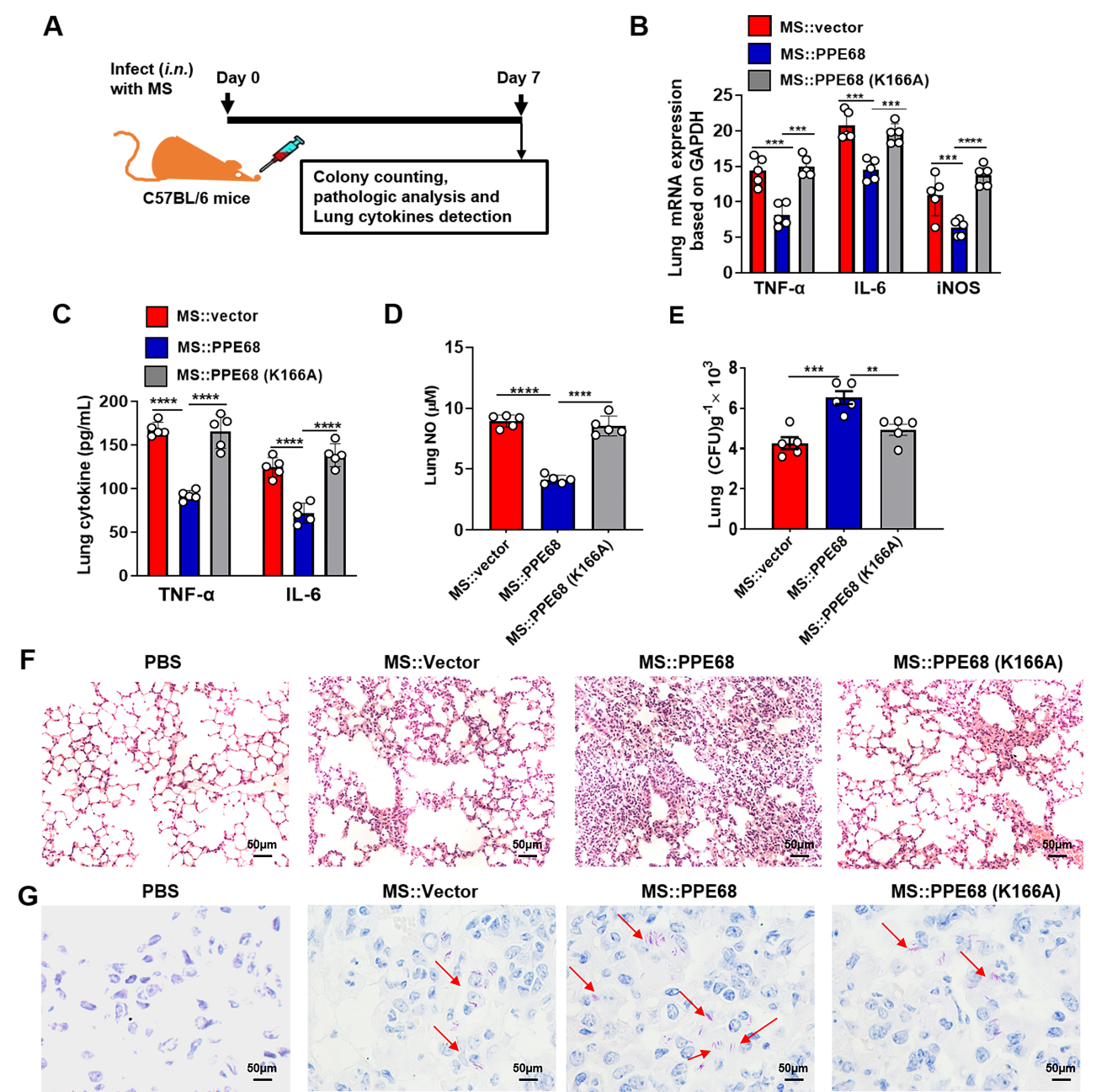
**

**Supplementary Figure 6. PPE68 inhibits the production of TNF-α, IL-6 and NO and promotes bacterial survival in macrophages via its K166 ubiquitination in mice. (A)** Procedure for the mouse MS infection experiment. **(B)** RT-qPCR analysis of lung TNF-α, IL-6 and iNOS mRNA expression of the C57BL/6 mice infected (*i.n.*) with 10^7^ CFUs of MS::vector, MS::PPE68 or MS::PPE68 (K166) at day 7 post-infection. **(C-E)** Lung TNF-α, IL-6 **(C)** and NO **(D)** were detected, and bacterial burden in the lungs was determined by CFU counting on 7H10 agar plates **(E)** at day 7 post-infection. Lung tissue sections were analyzed with H&E **(F)** and acid-fast staining **(G)**. The red arrows indicate acid-fast staining-positive bacteria. The data are expressed as the mean ± SD of three independent experiments for **B-E**, two tailed unpaired t test was used to calculate statistical significance. ***p* < 0.01; ****p* < 0.005; *****p* < 0.0001.

**Supplementary Figure 7.**
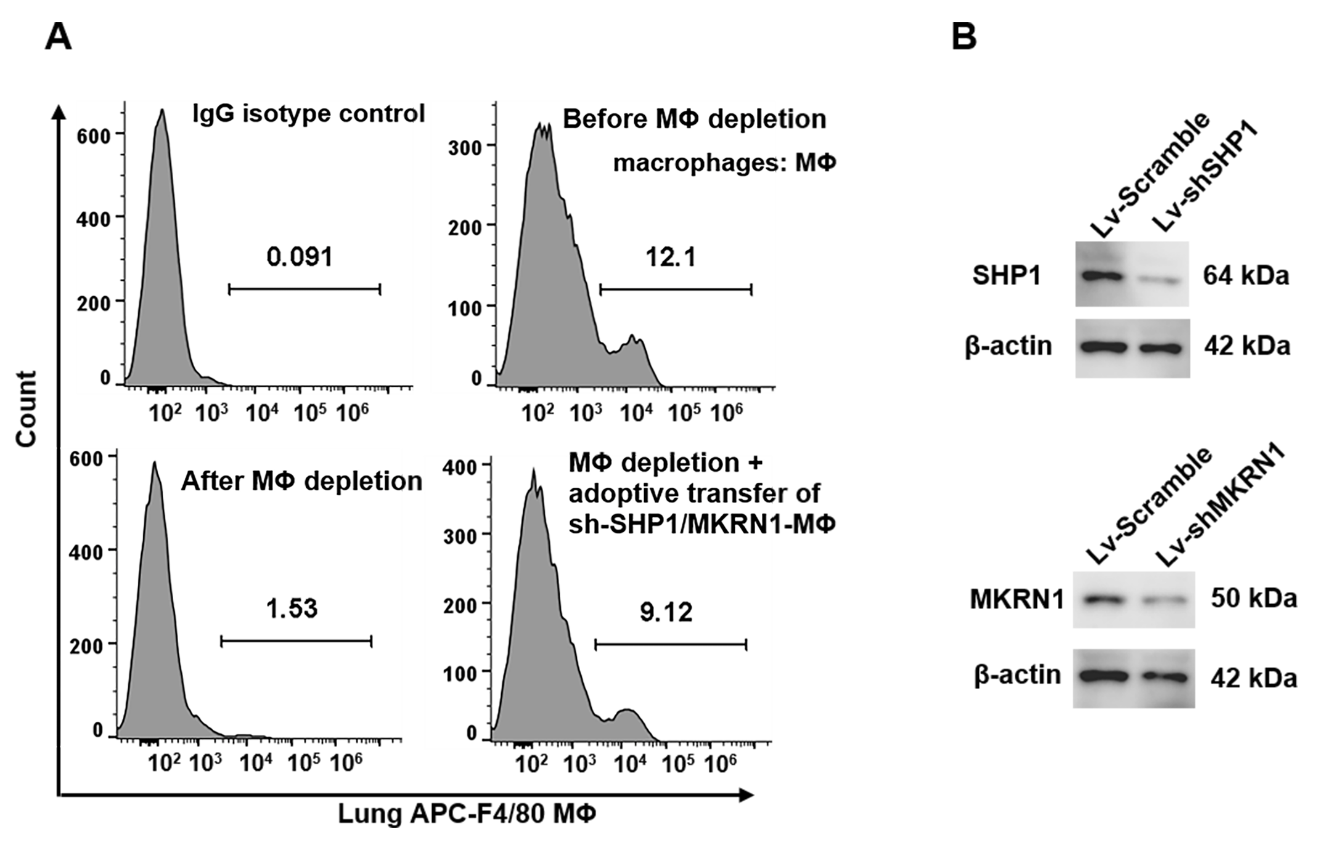


**Supplementary Figure 7. Assay of shSHP1/shMKRN1 knockdown efficiency and macrophage depletion/adoptive transfer efficiency.** (**A**) FCM analysis of macrophage depletion efficiency and adoptive transfer efficiency with an APC-labeled F4/80 antibody (murine macrophage marker). Top left, mouse IgG isotype control. Top right, lung macrophages (MΦ) before clodronate liposome treatment. Down left, lung MΦ after clodronate liposome treatment. Down right, lung MΦ after adoptive transfer of Lv-shSHP1/MKRN1 infected macrophages. (**B**) Western blot analysis of shSHP1 (up) and shMKRN1 (down) knockdown efficiency in BMDMs infected with the lentivirus scramble control (Lv-sh-Scramble), lentivirus containing shSHP1 (Lv-shSHP1) or lentivirus containing shMKRN1 (Lv-shMKRN1) by using anti-SHP1 or anti-MKRN1 antibodies.

**Supplementary Table 1. All primers and plasmids used in this study**

| **Primer names** | **Primer sequences (5'-3')** |
| --- | --- |
| PPE68-F  PPE68-R | GCGGATCCATGCTGTGGCACGCAATGCC  ACAAGCTTTCACCAGTCGTCCTCTTCGT |
| PPE68 (K63R) | GAGGTGGCAGCGACAGCGCGCTTGCGGCTGCAA  TTGCAGCCGCAAGCGCGCTGTCGCTGCCACCTC |
| PPE68 (K83R) | GCGTCAACACAGGCCGCGACCCGTGCGATGCAG  CTGCATCGCACGGGTCGCGGCCTGTGTTGACGC |
| PPE68 (K166R) | AACACGCTTTTCGAGGCGCTCGAGCCGATGGCG  CGCCATCGGCTCGAGCGCCTCGAAAAGCGTGTT |
| PPE68 (K302R) | TCTCAGCTGATCGAAGCGCCGGTTGCCCCCTCG  CGAGGGGGCAACCGGCGCTTCGATCAGCTGAGA |
| TLR2 (human)-F  TLR2 (human)-R | CTGCGGCCGCGATGCTACGAGCTCTTTGGCT  CTTCTAGAGGACTTTATTGCAGTTCTCA |
| MyD88 (human)-F  MyD88 (human)-R | GCTAAGCTTATGCGACCCGACCGCGCTGA  GCTTCTAGATCAGGGCAGGGACAAGGCCT |
| MKRN1 (human)-F  MKRN1 (human)-R | CTGAATTCATGGCGGAGGCTGCAACTCCCGG  GTAAGCTTTAGATCCAAGTCATAAAAATCTTCC |
| M1 (human)-F  M1 (human)-R | GTGAATTCATGGCAACTGTAGGAGCAGGTTC  GTAAGCTTCTAGATTACAAGGATGACGACGATAAGTAGATCCAAGTCATAAAAATCT |
| M2 (human)-F  M2 (human)-R | GTGAATTCATGTGCCCCTATGCTGCAGTGGG  GTAAGCTTCTAGATTACAAGGATGACGACGATAAGTAGATCCAAGTCATAAAAATCT |
| M3 (human)-F  M3 (human)-R | GTGAATTCATGAGGTATTTTGATGAAGGACG  GTAAGCTTCTAGATTACAAGGATGACGACGATAAGTAGATCCAAGTCATAAAAATCT |
| COPS5 (human)-F  COPS5 (human)-R | CTGAATTCATGGCGGCGTCCGGGAGCGGTAT  GTAAGCTTTTAAGAGATGTTAATTTGATTAA |
| SHP1 (human)-F  SHP1 (human)-R | ATGAATTCTACCCATACGATGTTCCAGATTACGCTATGTTGTCCCGCGGGTGGTT  ATAAGCTTTCACTTCCTCTTGAGAGAAC |
| TNF-α (mouse)-F  TNF-α (mouse)-R | AGGCACTCCCCCAAAAGATG  CCACTTGGTGGTTTGTGAGTG |
| IL-6 (mouse)-F  IL-6 (mouse)-R | TGTCTATACCACTTCACAAGTCGGAG  GCACAACTCTTTTCTCATTTCCAC |
| iNOS (mouse)-F  iNOS (mouse)-R | AATAGAGGAACATCTGGCCAGG  ATGGCCGACCTGATGTTGC |
| GAPDH (mouse)-F  GAPDH (mouse)-R | ACCACAGTCCATGCCATCAC  TCCACCACCCTGTTGCTGTA |
| Rv3872-F  Rv3872-R | AAGTGAGCGACAACGCTCTG  TCGTCGATTTGCGAATAGGT |
| Rv3873-F  Rv3873-R | GCTGATGTCTCAGCTGATCG  GTCGTCTTCTTCACGCTCCT |
| Rv3874-F  Rv3874-F | GCAGAGATGAAGACCGATGC  GCTTATTGGCTGCTTCTTGG |
| 16sRNA-F  16sRNA-R | CCGCGGCCTATCAGCTTGTTGGT  GTAGTTGGCCGGTGCTTCTTCTCC |
| Mouse MKRN1 | gRNA-A1: TACTTGCACACTACGCCGTA-TGG  gRNA-B1: CTTTATGCATGGGGTTTGTA-AGG  gRNA-C1: TCTTACAGATACTTTATGCA-TGG |
| Mouse SHP-1 | gRNA-A1: TGGTACCACGGCCACATATC-TGG  gRNA-B1: TACCACGGCCACATATCTGG-AGG  gRNA-C1: AAATGTCCAGGGCTCGCCCT-TGG |
| sh-SHP1 (mouse)-1 | AGGAGTTTGAGAGTCTACAAAAGCTCGAGCTTTTGTAGACTCTCAAACTCCTTTTT (sense)  AATTAAAAAAGGAGTTTGAGAGTCTACAAAAGCTCGAGCTTTTGTAGACTCTCAAACTCCGGCC (anti-sense) |
| sh-SHP1 (mouse)-2 | GTCATTGATATGCTTATGGAAAGCTCGAGCTTTCCATAAGCATATCAATGACTTTTT (sense)  AATTAAAAAGTCATTGATATGCTTATGGAAAGCTCGAGCTTTCCATAAGCATATCAATGACGGCC (anti-sense) |
| sh-TRAF6 (mouse)-1 | GCAAAGCGAGAGATTCTTTCCCTCGAGGGAAAGAATCTCTCGCTTTGCTTTTT (sense)  AATTAAAAAGCAAAGCGAGAGATTCTTTCCCTCGAGGGAAAGAATCTCTCGCTTTGCGGCC (anti-sense) |
| sh-TRAF6 (mouse)-2 | GGACCCAAATTATGAGGAAACCTCGAGGTTTCCTCATAATTTGGGTCCTTTTT (sense)  AATTAAAAAGGACCCAAATTATGAGGAAACCTCGAGGTTTCCTCATAATTTGGGTCCGGCC (anti-sense) |
| sh-MKRN1 (mouse)-1 | GGCGAAGCTGAGTCACGAAACCTCGAGGTTTCGTGACTCAGCTTCGCCTTTTT (sense)  AATTAAAAAGGCGAAGCTGAGTCACGAAACCTCGAGGTTTCGTGACTCAGCTTCGCCGGCC (anti-sense) |
| sh-MKRN1 (mouse)-2 | AGCAATTTGAGAGCAAGATCACTCGAGTGATCTTGCTCTCAAATTGCTTTTTT (sense)  AATTAAAAAAGCAATTTGAGAGCAAGATCACTCGAGTGATCTTGCTCTCAAATTGCTGGCC (anti-sense) |
| sh-MKRN1(human)-1 | GCTGAGTCAAGAAATTCAAACCTCGAGGTTTGAATTTCTTGACTCAGCTTTTT (sense)  AATTAAAAAGCTGAGTCAAGAAATTCAAACCTCGAGGTTTGAATTTCTTGACTCAGCGGCC (anti-sense) |
| sh-MKRN1 (human)-2 | GCTGAGTCAAGAAATTCAAACCTCGAGGTTTGAATTTCTTGACTCAGCTTTTT (sense)  AATTAAAAAGCTGAGTCAAGAAATTCAAACCTCGAGGTTTGAATTTCTTGACTCAGCGGCC (anti-sense) |
| Scramble | ACGATAAGAAGTGTTGAAACCTCGAGACGATAAGAAGTGTTGAAACTTTTTT (sense)  AATTAAAAAACGATAAGAAGTGTTGAAACCTCGAGACGATAAGAAGTGTTGAAACGGCC (anti-sense) |
| pLKO.1-EGFP-shRNA-SHP-1 (mouse) | CCGGTTCTTGTAGCGGTTCTTGCTCTCAAGAGGCAAGAACCGCTACAAGAACATTTTTG  AATTCAAAAATTCTTGTAGCGGTTCTTGCTC  CTCTTGAGCAAGAACCGCTACAAGAACA |
| pLKO.1-EGFP-shRNA-MKRN1 (mouse) | CCGGGCTGAGTCAAGAAATTCAAACTCAAGAGTTGAATTTCTTGACTCAGCTTTTTTTG  AATTCAAAAAAAGCTGAGTCAAGAAATTCAACTCTTGAGTTTGAATTTCTTGACTCAGC |

**Supplementary Table 2.** **All plasmids used in this study**

| **Recombinant plasmids** | **SOURCE** | **IDENTIFIER** |
| --- | --- | --- |
| pcDNA3.1-Myc-PPE68 | This paper | N/A |
| pcDNA3.1-Myc-PPE68- K63A | This paper | N/A |
| pcDNA3.1-Myc-PPE68- K83A | This paper | N/A |
| pcDNA3.1-Myc-PPE68- K166A | This paper | N/A |
| pcDNA3.1-Myc-PPE68- K302A | This paper | N/A |
| pET-28a-PPE68 | This paper | N/A |
| pMV261-PPE68 | This paper | N/A |
| pMV261-PPE68-K166A | This paper | N/A |
| pcDNA3.1-Flag-TLR2 | This paper | N/A |
| pcDNA3.1-Flag-MyD88 | This paper | N/A |
| pcDNA3.1-Flag-COPS5 | This paper | N/A |
| pcDNA3.1-Flag-MKRN1 | This paper | N/A |
| pSilencer 1.0-U6-MKRN1 | This paper | N/A |
| pcDNA3.1-Flag-Smurf1 | This paper | N/A |
| pcDNA3.1-HA-SHP1 | This paper | N/A |
| pSilencer 1.0-U6-SHP1 | This paper | N/A |
| pLKO.1-EGFP-shRNA-SHP-1 | This paper | N/A |
| pLKO.1-EGFP-shRNA-MKRN1 | This paper | N/A |
| pcDNA3.1-Flag-TRAF6 | Drs. Xuetao Cao and Baoxue Ge | N/A |
| pcDNA3.1-Flag-TAK1 | Drs. Xuetao Cao and Baoxue Ge | N/A |
| pcDNA3.1-Flag-TAB1 | Drs. Xuetao Cao and Baoxue Ge | N/A |
| pcDNA3.1-Flag-TAB2 | Drs. Xuetao Cao and Baoxue Ge | N/A |
| pcDNA3.1-Flag-TAB3 | Drs. Xuetao Cao and Baoxue Ge | N/A |
| pcDNA-HA-Ub | Drs. Xuetao Cao and Baoxue Ge | N/A |
| pcDNA-HA-Ub(K6) | Drs. Xuetao Cao and Baoxue Ge | N/A |
| pcDNA-HA-Ub(K11) | Drs. Xuetao Cao and Baoxue Ge | N/A |
| pcDNA-HA-Ub(K27) | Drs. Xuetao Cao and Baoxue Ge | N/A |
| pcDNA-HA-Ub(K29) | Drs. Xuetao Cao and Baoxue Ge | N/A |
| pcDNA-HA-Ub(K33) | Drs. Xuetao Cao and Baoxue Ge | N/A |
| pcDNA-HA-Ub(K48) | Drs. Xuetao Cao and Baoxue Ge | N/A |
| pcDNA-HA-Ub(K63) | Drs. Xuetao Cao and Baoxue Ge | N/A |

**Supplementary Table 3 All Reagents used in this study**

| **REAGENT or RESOURCE** | **SOURCE** | **IDENTIFIER** |
| --- | --- | --- |
| **Antibodies** |  |  |
| Rabbit anti-p-P65 Antibody | Cell Signaling  Technology | Cat. No. 3033, RRID: AB_331284 |
| rabbit anti-p-IκBα Antibody | Cell Signaling  Technology | Cat. No. 2859, RRID: AB_561111 |
| rabbit anti-p-JNK Antibody | Cell Signaling  Technology | Cat. No. 4668, RRID: AB_823588 |
| rabbit anti-p-P38 Antibody | Cell Signaling  Technology | Cat. No. 4511, RRID: AB_2797648 |
| rabbit anti-p-ERK Antibody | Cell Signaling  Technology | Cat. No. 4376, RRID: AB_331772 |
| rabbit anti-p-Fos Antibody | Cell Signaling  Technology | Cat. No. 5348, RRID: AB_10557109 |
| rabbit anti-p-c-Jun Antibody | Cell Signaling  Technology | Cat. No. 3207, RRID: AB_2129575 |
| rabbit anti-TRAF6 Antibody | Cell Signaling  Technology | Cat. No. 8028,  RRID: AB_10858223 |
| Alexa Fluor 488-labelled anti-mouse Antibody | Cell Signaling  Technology | Cat. No. 4408, RRID: AB_2130165 |
| Alexa Fluor 594-labelled anti-rabbit Antibody | Cell Signaling  Technology | Cat. No. 8889, RRID: AB_2716249 |
| anti-Myc mAb | OriGene Technologies, Inc. | Cat. No. TA150121 |
| anti-MKRN1 mouse mAb | OriGene Technologies, Inc. | Cat. No. CF504092 |
| Rabbit anti-HA-Tag Antibody | ABclonal Biotech | Cat. No. AE036 |
| rabbit anti-iNOS Antibody | ABclonal Biotech | Cat. No. A0312 |
| mouse anti-GAPDH Antibody | ABclonal Biotech | Cat. No. AC002 |
| Rabbit anti-SHP1 Antibody | Proteintech Group | Cat. No. 24546-1-AP |
| mouse anti-β-actin Antibody | Proteintech Group | Cat. No. 66009-1-Ig |
| Mouse anti-Flag-Tag Antibody | Dia-An Biotech | Cat. No. 2064 |
| Mouse anti-PPE68 antibody | Dia-An Biotech | N/A |
| **Bacterial strains** |  |  |
| *Mtb* H37Rv | ATCC | ATCC 93009 |
| *Mtb* H37RvΔPPE68 | This paper | N/A |
| *Mtb* H37RvΔPPE68::PPE68 | This paper | N/A |
| MS | ATCC | ATCC 700084 |
| MS::PPE68 | This paper | N/A |
| MS::PPE68(K166) | This paper | N/A |
| *M. intracellulare* | ATCC | ATCC 13950 |
| *M. avium* | ATCC | ATCC 25291 |
| *M. marinum* | ATCC | ATCC 927 |
| *Escherichia coli* (*E. coli)* DH5α | ATCC | ATCC 25922 |
| *E.coli* BL-21 | ATCC | ATCC BAA-1025 |
| **Chemicals, Peptides, and**  **Recombinant Proteins** |  |  |
| Middlebrook 7H9 broth | BD Difco^TM^ | Cat. No. 271310 |
| Middlebrook 7H10 agar | BD Difco^TM^ | Cat. No. 262710 |
| BD BBL™ Middlebrook  OADC Enrichment | BD Difco^TM^ | Cat. No. 212351 |
| DAPI | Sigma-Aldrich | Cat. No. D9542 |
| Fetal bovine serum (FBS) | Gibco | Cat. No. 10099141 |
| M-CSF | Peprotech | Cat. No. 315-02-50 |
| Gentamicin | Sigma-Aldrich | Cat. No. E003632 |
| Lipopolysaccharide (LPS) | Sigma-Aldrich | Cat. No. L2630 |
| Protein A/G magnetic beads | MCE | Cat. No. HY-K0202 |
| NEOFECT™ DNA transfection reagent | Neofect Biotech | Cat. No. TF201201 |
| jetPEI®-Macrophage | Polyplus Transfection | Cat. No. 103-05N |
| PMSF | Roche | Cat. No. 10837091001 |
| TRIzol Reagent | Invitrogen | Cat. No. 15596026 |
| ReverTra Ace® qPCR RT Kit | Toyobo Life Science | Cat. No. FSQ-101 |
| SYBR® Green Realtime PCR  Master Mix | Toyobo Life Science | Cat. No. QPK-201 |
| Liposomes and clodronate liposomes (CLs) | FormuMax | Cat. No. F70101C-AC-10 |
| **Critical Commercial Assays** |  |  |
| Mouse TNF-α ELISA kit | Dakewe Biotech | Cat. No. 1210122 |
| Mouse IL-6 ELISA kit | Dakewe Biotech | Cat. No. 1210122 |
| NO assay kit | Beyotime | Cat. No. S0024 |
| **Cell lines** |  |  |
| RAW264.7 | The China Center for Type Culture  Collection (CCTCC) | Cat. No. GDC0143 |
| HEK293T | The China Center for Type Culture  Collection (CCTCC) | Cat. No. ACS-4500 |
| **Animals** |  |  |
| Mouse: C57BL/6 | Animal Laboratory  Center of Wuhan  University | http://shydw.whu.edu.cn/ |

**Supplementary Table 4. All softwares used in this study**

| Software and Algorithms |  |  |
| --- | --- | --- |
| GraphPad Prism | GraphPad Prism software | https://www.graphpad.com/ |
| Image J | Image J software | https://imagej.en.softonic.com/ |
| AlphaFold Protein Structure Database | Protein structure prediction | https://alphafold.ebi.ac.uk/ |
